# Patient-reported outcomes in refractory hormone-producing pituitary adenomas: an unmet need

**DOI:** 10.1007/s11102-023-01309-4

**Published:** 2023-04-04

**Authors:** Victoria R. van Trigt, Iris C. M. Pelsma, Nienke R. Biermasz

**Affiliations:** Department of Medicine, Division of Endocrinology, and Center for Endocrine Tumors Leiden, Leiden University Medical Center, Albinusdreef 2, 2333 ZA Leiden, The Netherlands

**Keywords:** Pituitary adenomas, Refractory adenomas, Functioning adenomas, Patient-reported outcome measure, Health-related quality of life, Quality of reporting

## Abstract

**Purpose:**

To describe quality and outcomes of patient-reported outcome (PRO) measures (PROMs) used in patients with refractory hormone-producing pituitary adenomas, and to provide an overview of PROs in these challenging pituitary adenomas.

**Methods:**

Three databases were searched for studies reporting on refractory pituitary adenomas. For the purpose of this review, refractory adenomas were defined as tumors resistant to primary therapy. General risk of bias was assessed using a component approach and the quality of PROM reporting was assessed using the International Society for Quality of Life Research (ISOQOL) criteria.

**Results:**

20 studies reported on PROMs in refractory pituitary adenomas, using 14 different PROMs, of which 4 were disease specific (median general risk of bias score: 33.5% (range 6–50%) and ISOQOL score: 46% (range 29–62%)). SF-36/RAND-36 and AcroQoL were most frequently used. Health-related quality of life in refractory patients (measured by AcroQoL, SF-36/Rand-36, Tuebingen CD-25, and EQ-5D-5L) varied greatly across studies, and was not always impaired compared to patients in remission.

**Conclusion:**

There is a scarcity of data on PROs in the subset of pituitary adenomas that is more difficult to treat, e.g., refractory and these patients are difficult to isolate from the total cohort. The patients' perspective on quality of life, therefore, remains largely unknown in refractory patients. Thus, PROs in refractory pituitary adenomas require adequate analysis using properly reported disease specific PROMs in large cohorts to enable appropriate interpretation for use in clinical practice.

**Supplementary Information:**

The online version contains supplementary material available at 10.1007/s11102-023-01309-4.

## Introduction

The definition of refractory hormone-producing pituitary adenomas is ambiguous. Moreover, ‘refractory tumors’ or refractoriness was not defined in the 4th edition of the World Health Organization Guidelines for Classification of Pituitary Tumors [1]. Throughout the current literature, multiple definitions have therefore been used depending on the type of pituitary adenoma: adenomas not responding to conventional doses of dopamine agonists (DAs) in prolactinomas [2–4], failure of pituitary tumor resection or radiotherapy (RT) in Cushing’s Disease (CD) [5], and a combination of (a) Ki-67 index > 3%, (b) > 2% monthly growth, (c) resistance to current treatments and (d) recurrence ≤ 6 months after surgery for all pituitary adenomas [6].

Regardless of the exact definition, refractoriness can theoretically result in prolonged treatment, more interventions, higher disease burden, longer exposure to supraphysiological hormone levels, and higher risk of hypopituitarism. Therefore, refractory patients might be more prone to impaired quality of life (QoL) and functional disability compared to patients with pituitary tumors who are cured by a single intervention [7, 8]. Biochemical and other clinician reported outcomes, however, might be discordant with patient-reported health-related QoL (HR-QoL), and other patient-reported outcomes (PROs) in pituitary tumors [9–11]. Thus, clinician reported outcomes and PROs should be used simultaneously [12, 13].

Various generic, and disease-specific patient-reported outcome measures (PROMs) have been developed, which are increasingly being used in the field of pituitary care and research. Moreover, PROMs are used to classify patients holistically, e.g., using SAGIT and ACRODAT in patients with acromegaly [14, 15]. Despite the increased use of PROMs, no previous systematic review has focused on PROs in refractory pituitary adenomas. In this systematic review, quality and outcomes of PROMS used in patients with refractory hormone-producing pituitary adenomas are described.

## Materials and methods

This systematic review was performed in accordance with the Preferred Reporting Items for Systemic Reviews and Meta-Analyses (PRISMA) guidelines [16].

### Literature search and eligibility criteria

A literature search was conducted on 16-09-2022 (PubMed, Embase and Web of Science). The full search strategy, and in- and exclusion criteria are shown in Supplement 1 and 2, respectively. In brief, articles reporting on PROMs in patients with refractory hormone-producing pituitary adenomas in English were included. Articles were excluded if no full text was available, if they reported on < 5 refractory patients per disease, or on non-original data.

Following consensus amongst the authors, for this review, refractory adenomas were defined as *difficult-to-treat adenomas*, meeting the following criteria: hormone-producing adenomas not responding to first-line therapy—either pituitary surgery for acromegaly, CD, thyrotrophic adenomas (TSH-oma) and gonadotropinomas, or the maximum tolerated dose of DAs for prolactinomas. Studies on prolactinomas resistant to surgical treatment, and studies on pituitary adenomas for which surgery was the primary treatment option, but not performed in all patients (due to contraindications), were also included. Consequently, due to paucity of data, the definition of refractory adenoma was highly inclusive. Notably, no PRO studies on patients with aggressive pituitary tumors were available.

### Data extraction

All identified studies were imported into Endnote X9. Studies were screened by title and abstract and those of interest were reviewed by full-text screening. An overview of extracted data was shown in Supplement 3. If data was only presented in figures without absolute values, numerical values were estimated.

### PROMS

Questionnaires were the only type of PROMs used in the included articles, and therefore solely these results were reported. All PROMs were described briefly below and elaborately in Supplement 4.

#### Disease-specific

The validated Acromegaly Quality of Life Questionnaire (AcroQoL) assesses four domains of HR-QoL (range 0–100, with higher scores indicating better HR-QoL) [17]. Tuebingen Cushing’s Disease quality of life inventory (Tuebingen CD-25) and Cushing Quality of Life Questionnaire (CushingQoL), both validated in patients with CD, describe multiple dimensions of HR-QoL in CD (range 0–100, with higher scores indicating worse HR-QoL for Tuebingen CD-25 and better HR-QoL for CushingQoL) [18, 19]. Discomfort in acromegaly is quantified by Acromegaly Comorbidities & Complaints Questionnaire (ACCQ) (range 0–24, with higher scores indicating more discomfort) [20].

#### Pituitary specific

Pituitary Quality of Life Questionnaire (PIT QOL) describes HR-QoL in patients with pituitary disease (range 0–371, with higher scores indicating better HR-QoL [21]).

#### Generic HR-QoL

The 36-item short-form (SF-36) and Research and Development-36 (RAND-36) measure eight domains of HR-QoL and two component scales (range 0–100, with higher scores indicating better HR-QoL) [22, 23]. SF-12 is the shorter, 12-question version of this questionnaire [24]. EQ-5D-5L measures 5 health dimensions and includes a visual analogue score (VAS). Raw values can be transformed into index scores using population specific value sets (index score range 0.446–1.00, with higher scores indicating worse HR-QoL, VAS: 0–100, with higher scores indicating better HR-QoL). 15-Dementional (15-D) measures general HR-QoL (range 0–1, with higher scores indicating better HR-QoL) [25].

#### Symptom specific

Beck Depression Inventory (BDI) determines signs and intensity of depression (range 0–63, with higher scores indicating worse depression). The Multidimensional Body-Self Relations Questionnaire (MBSRQ) measures body satisfaction (range 0–5, with higher scores indicating more satisfaction)[26, 27]. SCL-90-R assesses nine domains of psychopathology (range 0–100, with higher scores indicating more distress or disturbance) [28]. Cloninger’s Tridimensional Personality Questionnaire (TPQ) measures *novelty seeking* (range 0–34), *harm avoidance* (range 0–34) an *reward dependence* (range 0–30), with higher scores indicating stronger emphasis on the behavior [29, 30]. Hospital Anxiety and Depression Scale (HADS) describes the severity of anxiety and depression in outpatient settings (range 0–21, with higher scores indicating more anxiety and depression) [31].

### Risk of bias assessment

The quality of selected articles was assessed using a component approach for the general risk of bias [32], and the quality of reporting on PROs by the modified ISOQOL criteria for non-randomized studies [33, 34] (Supplement 5). The cut-off for sufficient quality of reporting was 69%, as previously published [34, 35].

### Data analysis

Microsoft Excel (Microsoft Corporation, Redmond, WA, USA) was used for data collection. The primary study outcome were PROMs. The secondary outcomes were the quality of reporting on PROMs and PRO results. Statistical analysis could not be performed, due to insufficient data to perform a meta-analysis.

## Results

### Study selection

**Fig. 1:**
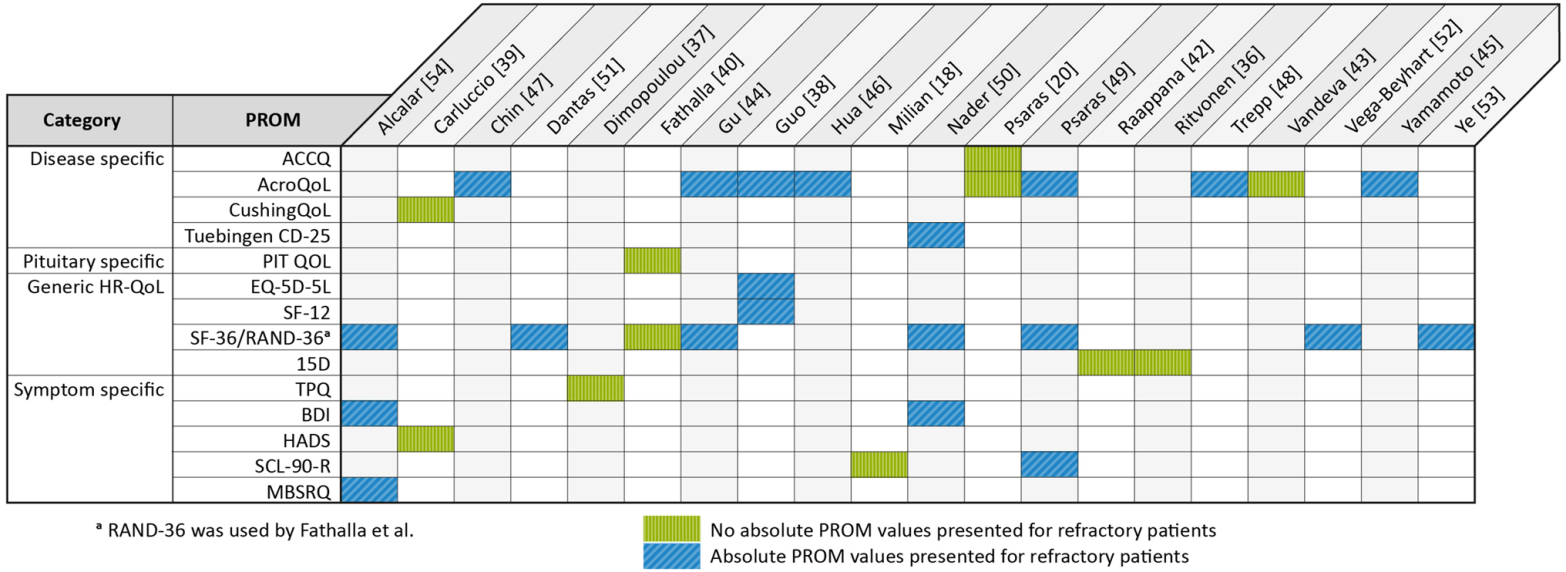
Patient reported outcome measures for refractory patients per study. *AcroQoL* Acromegaly Quality of Life Questionnaire, *ACCQ* Acromegaly Comorbidities & Complaints Questionnaire, *BDI* Beck Depression Inventory, *CushingQoL* Cushing Quality of Life Questionnaire, *EQ-5D-5L* 5-level EuroQoL-5, *HADS* Hospital Anxiety and Depression Scale, *MBSRQ* Multidimensional Body-Self Relations Questionnaire, *PIT QOL* Pituitary Quality of Life, *PROM* patient reported outcome measure, *SCL-90-R* Symptom Checklist-90-Revised, *SF-36* Short Form-36, *RAND-36* Research and Development-36, *TPQ* Cloninger’s Tridimensional Personality Questionnaire, *Tuebingen CD-25* Tuebingen Cushing’s Disease Quality of Life Inventory, *15D* 15-Dimentional

A total of 4554 articles were screened for eligibility, as depicted in the flowchart of article screening and inclusion in Supplement 6. Twenty articles were included in the systematic review, of which 14 were cross-sectional studies, 5 were cohort studies, and 1 article reported on cross-sectional and cohort data (study characteristics: Supplement 7). As some studies reported on multiple types of refractory adenomas, the number of studies reporting on patients with the included pituitary diseases were 14 for refractory acromegaly, 6 for refractory CD, and 4 for refractory prolactinoma. No studies reported on TSH-oma or gonadotropinoma. In total, 14 different PROMs reported on refractory adenomas, of which 4 were disease-specific (overview of PROMs per study: Fig. 1).

### Risk of bias assessment

Estimated risk of bias was high in all studies (median score 33.5%, range 6–50%) (Supplement 8). None of the studies defined refractoriness. Two studies explicitly stated that patients with active disease were symptomatic, although none included a definition of the symptoms. Four studies reported on missing PROM data, of which only one reported < 10% missing data [36]. Quality of PROMs reporting was insufficient in all studies (median score 46%, range 29–62%) (Supplement 9). Two studies included a hypothesis specifically for the used PROM [20, 37], whereas only one described the method of statistical analysis for the PROM hypothesis [37]. Solely one study described a statistical approach for missing PRO data [38].

### PROMs

AcroQoL and SF-36 were most frequently used. In 8/20 studies, absolute PRO results were not reported, with only conclusions being reported on whether refractory patients scored higher or lower than patients in remission or healthy controls [20, 36, 37, 39–43].

#### Disease-specific HR-QoL

##### AcroQoL

AcroQoL was used in nine studies (Fig. 1), of which seven reported absolute values. In these seven studies, scores of refractory acromegaly patients compared to patients in remission varied substantially, described as either comparable in the two patient groups in four studies [44–47], decreased in one study [38], or decreased except for the domain *personal relations* in another study [48] (Table 1). One study compared refractory acromegaly patients to healthy controls, finding lower scores in all domains for refractory patients [49]. By contrast, two studies did not present absolute values, of which one reported comparable scores in refractory patients compared to patients in remission [20], and the other reported that AcroQoL scores improved less after treatment (TSS/RT/DA) in refractory patients compared to patients in remission [43] (mean follow-up time: 29.6 ± 19.7 months and 29.3 ± 18.8, respectively).

**Table 1:** AcroQoL scores per study

	Chin [47]	Gu [44]	Guo [38]	Hua [46]		Psaras [49]	Trepp [48]	Yamamoto [45]^f^	
Baseline	Mean (range) N = 36	Median [IQR] N = 44	Mean ± SD N = 154	Mean ± SD N = 11	Mean ± SD N = 11	Mean ± SD N = 14	Mean ± SD N = 6	Median [IQR] N = 20	Median [IQR] N = 18
	Before SMS	Preoperative		SMS ( +)^a^	SMS (−)			< 65 years-old	≥ 65 years-old
Physical	67.5 (30.0–97.5) =^c^		41.3 ± 25.5 ↓	55.1 ± 30.4^d^	42.3 ± 33.2^d^	67.7 ± 22.4^e^	42 ± 32↓	70 [31–82] =	52 [32–80] =
Psychological	74.3 (27.1–91.4) = ^c^		40.1 ± 22.9 ↓	55.7 ± 24.8^d^	56.4 ± 29.4^d^	62.5 ± 20.2^e^	44 ± 23↓	61 [50–76] =	67 [50–85] =
*Appearance*^*b*^	65.7 (22.9–88.6) = ^c^		33.9 ± 21.5 ↓	47.4 ± 26.2^d^	43.8 ± 34.5^d^	51.8 ± 25.3^e^	30 ± 21↓		
*Personal relations*^*b*^	82.9 (31.4–100.0) = ^c^		46.3 ± 26.9 ↓	65.9 ± 24.2^d^	69.2 ± 28.7^d^	77.3 ± 17.6^e^	52 ± 24 =		
Total	74.6 (33.6–93.6) = ^c^	64.1 [51.8–71.8] =	40.5 ± 22.9 ↓	56.1 ± 26.4^d^	51.3 ± 28.6^d^	64.9 ± 17.8^e^	43 ± 25↓	67 [42–78] =	61 [43–85] =
First follow-up	12 weeks After starting SMS^g^	6 months postoperative							
Physical	71.3 (40.0–97.5) =^c^								
Psychological	78.6 (27.1–97.1) = ^c^								
*Appearance*^*b*^	72.3 (22.9–94.3) =^c^								
*Personal relations*^*b*^	80.0 (31.4–100.0) = ^c^								
Total	74.1 (32.7–99.5) = ^c^	82.7 [74.1–88.6] =							
Second Follow-up	24 weeks After starting SMS^g^								
Physical	71.3 (42.5–97.5) = ^c^								
Psychological	78.6 (27.1–97.1) = ^c^								
*Appearance*^*b*^	77.1 (22.9–97.1) =^c^								
*Personal relations*^*b*^	85.7 (21.4–100.0) = ^c^								
Total	77.3 (32.7–95.5) = ^c^								

AcroQoL scores for refractory patients with acromegaly per study**.** *AcroQoL* Acromegaly Quality of Life Questionnaire, *IQR* interquartile range, *SMS(* +*)* on somatostatin analogue treatment, *SMS(−)* not on somatostatin analogue treatment, *↓* significantly lower compared to acromegaly patients in remission; *↑* significantly higher compared to acromegaly patients in remission, no P-value reported, = tested and no significant difference compared to patients in remission

^a^Patients received octreotide LAR every two weeks (dose not reported)

^b^Psychological subscales

^c^AcroQoL scores did not differ between refractory patients and patients in remission at 24 weeks

^d^No significant difference between all refractory patients SMS (+) or SMS (−) and patients in remission. No subgroup analysis performed for SMS(+) and SMS(−) separately

^e^Refractory patients scored significantly lower than healthy controls

^f^Values estimated based on figure, absolute values were not presented

^g^Patients received weekly intramuscular injections of octreotide LAR 20 mg. At 12 weeks a dose escalation to octreotide LAR 30 mg was permitted in case GH > 2.5 ug/L and/or IGF1 above upper limit of normal for age, but this was not obligatory

##### ACCQ

One study reported on the ACCQ in refractory acromegaly, albeit without presenting absolute values, and concluded the scores were comparable to patients in remission [20].

##### Tuebingen* CD-25*

Tuebingen CD-25 scores of refractory CD patients were reported in one study, showing no difference with CD patients in remission [50](Supplement 10).

##### CushingQoL

The only study reporting on the CushingQoL reported lower (i.e., worse) CushingQoL scores in refractory CD patients compared to patients in remission [39]. Absolute values were not presented.

##### *PIT *QOL

PIT QOL scores, solely reported in one study and without presenting absolute values, were comparable in refractory acromegaly patients and patients in remission [40].

#### Generic HR-QoL

##### SF 12/36 and RAND-36

SF-12/36 and RAND-36 were reported in nine studies (Table 2), of which eight presented absolute values. The results were inconsistent across studies and between diseases. In acromegaly patients, one study reported comparable results between refractory acromegaly patients and patients in remission [44], one reported lower scores in refractory patients except for *physical functioning* and *general health* [38] and another reported lower scores in refractory acromegaly only in the *role physical, bodily pain* and *vitality* domains [51]. The study that did not report absolute values found no difference in RAND-36 scores between refractory acromegaly and patients in remission [40].

**Table 2:** SF-12 and SF-36 scores per study

First Author, year of publication	Dantas [51]	Gu [44]	Guo [38] SF-12	Psaras [49]	Alcalar [54]	Nader [50]^d^	Psaras [49]	Vega-Beyhart [52]	Ye [53]^d^	Vega-Beyhart [19] 52
Disease	AC	AC	AC	AC	CD	CD	CD	CD	CD	PRL
Baseline	Mean N = 14^a^	Median [IQR] N = 44	Mean ± SD N = 154	Mean ± SD N = 14	Mean ± SD N = 8	Unclear^e^ N = 8	Mean ± SD N = 5	Median [IQR] N = 7	Unclear^g^ N = 7	Median [IQR] N = 28
Physical functioning	54.09 =		49.1 ± 9.3 =	51.0 ± 25.8	18.63 ± 5.61^c^	75 = ^f^	37.6 ± 32.4	45 [20–85]↓	55 [52–60]	82 [48–95]↓
Role physical	50.00↓		40.9 ± 11.4↓	43.2 ± 29.0	5.63 ± 1.99	75 =^f^	25.0 ± 23.1	25 [0–50]↓	29 [23–32]	50 [0–100]↓
Bodily pain	43.64↓		36.7 ± 11.7↓	35.7 ± 20.6	6.81 ± 3.52^c^	25 =^f^	44.6 ± 24.2	45 [25–67]↓	58 [54–62]	78 [45–90] =
General health	63.36 =		32.0 ± 10.7 =	39.9 ± 29.7^b^	11.88 ± 3.68^c^	38 =^f^	39.7 ± 37.8^b^	40 [20–45] =	34 [30–39]	48 [26–65]↓
Social functioning	60.00 =		38.3 ± 12.1↓	43.4 ± 34.7	7.00 ± 2.14	62 =^f^	31.6 ± 32.9^b^	50 [37–62]↓	50 [45–55]	62 [40–75]↓
Role emotional	45.27 =		35.1 ± 12.8↓	39.7 ± 31.3^b^	4.25 ± 1.28	62 =^f^	26.0 ± 29.3^b^	0 [0–0]↓	38 [32–41]	50 [0–100] ↓
Mental health	66.91 =		37.2 ± 5.6↓	38.2 ± 37.2^b^	20.00 ± 5.90	62 =^f^	43.8 ± 42.7^b^	44 [16–67]↓	56 [50–60]	56 [36–68]↓
Vitality	45.45↓		44.7 ± 10.6↓	33.8 ± 29.4^b^	13.63 ± 5.15	50 =^f^	47.2 ± 38.0	45 [10–60] =	32 [28–35]	45 [35–67]↓
MCS			38.9 ± 8.0↓					29 [22–53]↓		57 [31–69]↓
PCS			39.6 ± 8.8↓					44 [16–70]↓		66 [36–81]↓
Total		65.4 [63.2–67.7] =								
First follow-up		6 mos postop							Mean 2.35 mos postop, N = 6^ h^	
Physical functioning									49 [42–52]	
Role physical									23 [19–30]	
Bodily pain									65 [60–70]	
General health									28 [22–34]	
Social functioning									53 [50–69]	
Role emotional									45 [40–50]	
Mental health									51 [45–59]	
Vitality									25 [20–30]	
Total		75.3 [70.1–82.3] =								
Second follow-up									Mean 7.4 mos postop, N = 4^ h^	
Physical functioning									50 [45–54]	
Role physical									30 [27–34]	
Bodily pain									60 [66–64]	
General health									42 [60–47]	
Social functioning									61 [58–65]	
Role emotional									58 [52–61]	
Mental health									55 [50–60]	
Vitality									39 [35–42]	

SF-12 and SF-36 scores for refractory patients with acromegaly, Cushing’s Disease and prolactinoma per study. *AC* acromegaly, *CD* Cushing’s Disease, *IQR* interquartile range, *MCS* mental component summary, *mos* months, *PCS* physical component summary, *postop* postoperative, *PRL* prolactinoma, *SD* standard deviation, *↓* significantly lower compared to patients in remission; *↑*significantly higher compared to patients in remission, no P-value reported, = tested and no significant difference compared to patients in remission

^a^Number of patients not reported in article. Author provided information upon request

^b^Refractory patients scored significantly lower than healthy controls

^c^Refractory patients scored significantly lower than healthy controls and patients in remission (no post-hoc analysis was performed)

^d^Values estimated based on figure, absolute values were not presented

^e^Unclear whether reported numbers concern mean or median values

^f^All SF-36 domain scores were lower in refractory patients compared to patients in remission, however not significant

^g^Unclear what the values indicate. Figure does not include a legenda

^h^Missing data was not reported in article. Author provided information upon request

In CD patients, one study reported comparable scores in refractory CD compared to patients in remission [50], and one reported lower scores except for the *general health* and *vitality* domains [52]. Furthermore, one study concluded no postoperative trend of improvement over time (mean 7.4 months) was observed in refractory CD patients, whereas CD patients in remission did improve postoperatively [53]. Two studies compared refractory CD patients to healthy controls, of which one found lower scores in refractory patients only for *physical functioning*, *bodily pain* and *general health* [54], and the other found lower scores for *general health*, *mental health*, *social functioning* and *role emotional* [49].

One study reported on SF-36 in refractory prolactinomas, finding lower scores compared to patients in remission except for the *bodily pain* domain [52].

##### EQ-5D-5L

EQ-5D-5L scales, reported in solely one study, for *pain/discomfort* and *anxiety/depression* were worse in refractory acromegaly patients compared to acromegaly patients in remission [38]. Mean EQ-5D VAS scores were 62.8 ± 21.6 in refractory acromegaly, which was similar compared to acromegaly in remission [38](Supplement 11).

##### 15D

Two studies reported on 15D without presenting absolute values, of which one on refractory acromegaly, CD and prolactinoma patients (without performing a subgroup analysis per disease) [36], and the other reported on refractory prolactinoma and acromegaly [42]. Both studies found comparable results in refractory patients compared to patients in remission.

#### Symptom-specific

##### BDI

BDI was reported in two studies, using different cut-off values. In refractory acromegaly, mean BDI scores were 18.9 ± 10.9 (Alcalar et al*.* used a score of > 17 points to indicate presence of depression) [54] (Supplement 12). In refractory CD, 2/8 patients scored ≥ 18 points (Nader et al*.* described a score of ≥ 18 points as a severe depression) [50].

##### SCL-90-R

Two studies reported on SCL-90-R. One found higher *hostility* scores in refractory AC and CD than in healthy controls, and *psychoticism* in refractory CD [49] (Supplement 13). The other, without presenting absolute values, reported higher *obsessive–compulsive* scores in refractory acromegaly patients compared to patients in remission 3 months after surgery, whereas no differences were observed at 12 months [41].

##### MBSRQ

Refractory CD patients had significantly lower MBSRQ scores for *fitness and health evaluation*, *body areas satisfaction* and *mean item score* compared to those in remission and healthy controls [54] (Supplement 14).

##### HADS

The only study reporting on HADS found higher anxiety scores in refractory CD patients compared to patients in remission [39]. Absolute values were not presented.

##### TPQ

TPQ, reported by only one study, without presenting absolute values, found higher *fear of uncertainty*, *fatigability* and *asthenia*, leading to a higher *total harm avoidance* score in refractory CD patients compared to CD patients in remission [37].

## Discussion

An unequivocal definition of refractory is lacking, and data, including patient-reported outcomes, on difficult-to-treat (e.g., refractory) patients is scarce. A plethora of PROMs were used in research and care of pituitary adenomas, of which few were disease specific. The quality of reporting in the available studies was low, with high risk of bias, leading to inconsistent PRO outcomes. Due to the paucity of data, no conclusions on HR-QoL and the contributing factors in refractory patients could be made.

Currently, no consensus on the definition of refractory is available in the literature, resulting in the application of the present definition (i.e., tumors not responding to primary therapy) for data selection. Using this definition, it should be noted that the status of refractoriness is not only dependent on tumor characteristics, but also on the surgical experience within the treating center, as more experienced surgeons may have somewhat better outcomes. However, from a patient's perspective, this definition might implicitly reflect the impact of the disease, due to prolonged absence of disease remission and the need for secondary treatment. Furthermore, the scarcity of data influenced the present, inclusive definition, as studies reporting on PROs in the most challenging patients (persistent disease despite multimodality treatment and aggressive tumors) were lacking. Consequently, these most challenging cases could not be identified at present, and therefore warrant future in-depth systematic investigation.

Nevertheless, there were some studies reported on PROs in patients with persistent disease after primary treatment—the present definition of refractory patients—to address our clinical question. The next challenge was the use of plethora of PROMs, which were mostly generic and sometimes disease-specific. Disease-specific PROMs focus on quality of life domains specifically impaired in the disease of interest, allowing identification of more subtle impairments than generic questionnaires [17, 55, 56].

Despite their better sensitivity, results of the disease specific questionnaires (ACCQ, AcroQoL, Tuebingen-CD25, CushingQoL) were equally ambiguous compared to those of the less sensitive, pituitary-specific (PIT QOL), and generic HQ-QoL questionnaires (EQ-5D-5L, SF12/36, RAND-36, 15D). Surprisingly, independent of the type of questionnaire used, results of refractory patients compared to patients in remission were inconsistent; being lower in some, yet comparable in other studies. A possible explanation may lie in the well-known fact that patients in remission report ongoing impaired quality of life.

Furthermore, there was no clear difference in outcomes between the types of adenomas. HR-QoL measured by SF-12/36 and RAND-36 varied greatly between the studies within same type of adenomas. Previous literature reported the worst HR-QoL in active CD compared to other pituitary adenomas [57], improving partially after remission [11, 58]. However, HR-QoL in refractory CD (measured by SF-12/36 or RAND-36) was not evidently lower than in other adenomas and not always worse compared to CD in remission. The symptom specific PROMS (HADS, TPQ, SCLR-90) found worse scores in varying—mostly psychological—domains, albeit inconsistent across studies. Similarly, in refractory acromegaly, subscales such as *bodily pain* and *physical functioning* (SF-12/36, RAND-36) and *appearance* (AcroQoL), expected to be most affected [7, 59], were not always worse compared to patients in remission. As expected, prolactinomas were the most understudied type of adenoma, with only one study reporting absolute values, thereby impeding proper comparison. Thus, overall results were inconsistent and inconclusive, regardless of questionnaire and adenoma type.

The well-known Wilson and Cleary model (WCM) [60] states that general wellbeing results from a complex interplay of physiological, clinical and social aspects. According to this model, HR-QoL can be influenced, either directly or indirectly, by six factors: biological and psychological factors, symptom status, functional status, general health perceptions and characteristics of the environment and of the individual. In patients with pituitary adenoma, irrespective of whether they are refractory, all these factors might be affected due to prolonged supraphysiological hormone levels, leading to severe symptomatology, decreased functional status, and impaired general health perceptions. Therefore, impaired HR-QoL may be anticipated in all patients with a pituitary tumor and the impact of having a more refractory status may be difficult to distillate from other factors influencing HR-QoL.

In agreement with the WCM, we found HR-QoL in refractory acromegaly and CD was not always worse than in patients in remission. This may be caused by ongoing symptoms in patients in biochemical remission, resulting from permanent complications (e.g., arthropathy in acromegaly and osteoporotic fractures and chronic depression in CD [61–64]) leading to persistently impaired HR-QoL. Contrarily, surgery could have improved symptomatology and functional status, thereby increasing HR-QoL, without achievement of biochemical remission in refractory patients [49]. However, true differences in HR-QoL may have been concealed by biased results, use of small sample sizes and generic questionnaires in the included studies. Furthermore, questionnaires cannot grasp all aspects of life.

The importance of the use of PROs in addition to clinician-reported outcomes is well recognized in care for pituitary disease, as well as other diseases [12, 13]. Ideally, PROs should focus on issues relevant to the specific (refractory) tumor, using a combination of generic, disease-specific and symptom-specific PROMs. Although consensus on which combination of PROMs to use is lacking, our group has gained some experience in selecting PROMs, according to the three-tier Value Based Health Care approach, at each relevant timepoint within the care trajectory [65]. This approach enables individualization of care trajectories. For this purpose, we developed the Leiden Bother and Needs Questionnaire, which is currently used in clinical practice to assess patients' bother related to consequences of the disease and their need for support [66]. An example of prospective PRO research including potentially difficult-to-treat (i.e., refractory) cases is the prolactinoma research project (PRolaCT) [67]. In the future, these care and research strategies should be used in patients with refractory adenomas.

An important limitation to this systematic review was the high risk of bias and low quality of PRO reporting, limiting proper interpretation and comparability. Secondly, isolating the patients who met our definition of refractory was challenging, as information on treatment was not always presented. This led to an inhomogeneous population. Due to the quality of data, no conclusions could be drawn about HR-QoL in refractory patients, compared to those in remission. Lastly, comparison of PROs with biochemical outcomes lay beyond the scope of this review, which would be interesting to place the PRO results in perspective. To adequately treat and support refractory patients, future studies using disease specific PROMs in large cohorts of patients with pituitary adenomas should be performed, with subgroup analyses for patients who are not in remission after primary therapy.

## Conclusion

The current systematic review demonstrated a scarcity of high-quality data on PROs in the subset of refractory pituitary adenomas—defined as adenomas being difficult to treat. Additionally, in the current literature, data from refractory patients was difficult to isolate from the rest of the cohort, and the patients' perspective on quality of life therefore remains largely unknown in refractory patients. Thus, PROs in patients with refractory hormone-producing pituitary adenomas require adequate analysis using properly reported disease-specific PROMs in large cohorts to enable appropriate interpretation and use for clinical practice.

## Supplementary Information

Below is the link to the electronic supplementary material.

Supplementary file1 (PDF 659 kb)

## Data Availability

Current manuscript includes no new data, all materials are included in our supplements.
