# Supplementary material for: Patient-reported outcomes in refractory hormone-producing pituitary adenomas: an unmet need
Source: Pituitary. 2023 Apr 4;26(3):307–17. doi: 10.1007/s11102-023-01309-4 (PMC10333395; doi:10.1007/s11102-023-01309-4)
Supplement: Supplementary file 1 — Supplementary file1 (PDF 659 kb) [file 11102_2023_1309_MOESM1_ESM.pdf]

Patient reported outcomes in refractory hormone producing pituitary adenomas: an unmet need

Supplements

Victoria R. van Trigt[1], Iris C.M. Pelsma[1], Nienke R. Biermasz[1]

Author affiliations

1. Dept. of Medicine, Division of Endocrinology, and Center for Endocrine Tumors Leiden, Leiden University Medical Center, Leiden, the Netherlands

Corresponding author:

V.R. van Trigt, MSc

Department of Medicine, Division of Endocrinology, Center for Endocrine Tumors Leiden,

Leiden University Medical Center,

Albinusdreef 2,

2333 ZA Leiden, Netherlands

Tel: 071-5263082

Email: V.R.van_Trigt@lumc.nl

Supplement 1 - Search strategy per database

Supplement 1a - PubMed

("Pituitary Neoplasms"[MeSH] OR "Pituitary Neoplasm*"[tiab] OR "Hyperprolactinemia"[MeSH] OR "Hyperprolactinemia"[tiab] OR "Hyperprolactinaemia"[tiab] OR "pituitary adenoma*"[tiab] OR "Prolactinoma"[MeSH] OR "Prolactinoma*"[tiab] OR "Microprolactinoma*"[tiab] OR "Macroprolactinoma*"[tiab] OR "Giant prolactinoma*"[tiab] OR "Pituitary Tumor*"[tiab] OR "hyperpituitarism"[MeSH] OR "acromegaly"[MeSH] OR "Acromegal*"[tiab] OR "gigantism"[MeSH] OR "Gigantism"[tiab] OR "growth hormone-secreting pituitary adenoma"[MeSH] OR "growth hormone-secreting pituitary adenoma"[tiab] OR "growth hormone secreting pituitary adenoma"[tiab] OR "pituitary acth hypersecretion"[MeSH] OR "pituitary acth hypersecretion"[tiab] OR "ACTH-Secreting Pituitary Adenoma*"[tiab] OR "Corticotroph Adenoma*"[tiab] OR "Cushing syndrome"[MeSH] OR "Cushing syndrome"[tiab] OR "Cushing's Syndrome"[tiab] OR "Hypercortisolism"[tiab] OR "Cushing disease"[tiab] OR "Cushing's disease"[tiab] OR "non-functioning adenoma*"[tiab] OR "non-functioning pituitary adenoma*"[tiab] OR "non-functioning macroadenoma*"[tiab] OR "nonfunctioning adenoma*"[tiab] OR "nonfunctioning pituitary adenoma*"[tiab] OR "nonfunctioning pituitary macroadenoma*"[tiab] OR "nonfunctioning macroadenoma*"[tiab] OR "nonfunctioning microadenoma*"[tiab]) AND ("Health Care Surveys"[MeSH] OR "Health Care Survey*"[tiab] OR "Patient Outcome Assessment"[MeSH] OR "Patient Outcome Assessment*"[tiab] OR "Quality of Life"[MeSH] OR "Quality of Life"[tiab] OR "Life Qualit*"[tiab] OR "Health-Related Quality Of Life"[tiab] OR "Health Related Quality Of Life"[tiab] OR "HR-QOL"[tiab] OR "Patient Reported Outcome Measure*"[tiab] OR "Patient Reported Outcome*"[tiab] OR "Patient-Reported Outcome*"[tiab] OR "Patient Outcome Assessment*"[tiab] OR "Patient-Centered Outcome*"[tiab] OR "Survey*"[tiab] OR "Questionnaire*"[tiab] OR "patient-reported symptom*"[tiab] OR "Patient Satisfaction"[MeSH] OR "Patient satisfaction"[tiab] OR "patient-reported experience measure"[tiab] OR "Patient experience"[tiab] OR "Health Care Survey*"[tiab] OR "Healthcare Survey*"[tiab] OR "Functional Status"[MeSH] OR "Functional Status"[tiab] OR "Health Behavior"[MeSH] OR "Health Behavior"[tiab] OR "Short-Form Health Survey*"[tiab] OR "Functional status"[tiab] OR "Health behavior*"[tiab] OR "Health-Related Behavior*"[tiab] OR "Health Related Behavior"[tiab] _OR "Self report*"[tiab] OR "Self-report*"[tiab] OR "Self-report"[tiab] OR "Self-reported"[tiab] OR "Outcome instrument*"[tiab] OR "Health scor*"[tiab] OR "Health status"[tiab] OR "Health outcome*"[tiab] OR "Observer-reported"[tiab] OR "Nurse-reported"[tiab] OR "Caregiver-reported"[tiab] OR "Caregiver-reported"[tiab] OR "Partner-reported"[tiab] OR "Subjective outcome*"[tiab] OR "SF36"[tiab] OR "SF-36"[tiab] OR "SF 36"[tiab] OR "EQ5D"[tiab] OR "EQ-5D"[tiab] OR "EQ 5D"[tiab] OR "EORTC"[tiab] OR "NHP"[tiab] OR "Nottingham health profile"[tiab] OR "LBNQ"[tiab] OR "Subjective wellbeing"[tiab] OR "Subjective well-being"[tiab] OR "sf-20"[tiab] OR "sf-6D"[tiab] OR "ghq-12"[tiab] OR "ghq-28"[tiab] OR "ghq-30"[tiab] OR "general health questionnaire"[tiab] OR "gwbs"[tiab] OR "general well-being scale"[tiab] OR "whoqol-bref"[tiab] OR "who-qol"[tiab] OR "World Health Organization Quality of Life Scale"[tiab] OR "sip"[tiab] OR "15D"[tiab] OR "SCL-90 (-R)"[tiab] OR "Symptom Checklist 90 (revised)"[tiab] OR "SRT"[tiab] OR "symptom rating test"[tiab] OR "ACROQoL"[tiab] OR "SSS"[tiab] OR "Quality of Life Questionnaire"[tiab] OR "SSS"[tiab] OR "PASQ"[tiab] OR "Patient-assessed-Acromegaly Symptom Questionnaire"[tiab] OR "QLS-H"[tiab] OR "QoL-AGHDA"[tiab] OR "HADS"[tiab] OR "Hospital Anxiety Depression Scale"[tiab] OR "MFI-20"[tiab] OR "Multidimensional Fatigue Inventory"[tiab] OR "MDI"[tiab] OR "Major Depression Inventory"[tiab] OR "NRS-pain"[tiab] OR "Numerical Rating Scale-pain"[tiab] OR "CFQ"[tiab] OR "Cognitive Failure Questionnaire"[tiab] OR "FACT"[tiab] OR "Functional Assessment of Cancer Therapy"[tiab] OR "Social Adjustment Scale"[tiab] OR "FSFI"[tiab] OR "Female Sexual Function Index"[tiab] OR "SSQ"[tiab] OR "Social Support Questionnaire"[tiab] OR "SQ"[tiab] OR "Symptom Questionnaire"[tiab] OR "BDI"[tiab] OR "Beck Depression Inventory"[tiab] OR "MBSRQ"[tiab] OR "Multidimensional Body-Self Relations Questionnaire"[tiab] OR "PSLES"[tiab] OR "Presumptive Stressful Life Events Scale"[tiab] OR "HIT-6"[tiab] OR "Headache Impact Test scale"[tiab] OR "CSCL"[tiab] OR "Coping Strategies Checklist"[tiab] OR "AIMS2"[tiab] OR "Arthritis Impact Measurement Scale 2"[tiab] OR "CPRS"[tiab] OR "Comprehensive Psychopathological Rating Scale"[tiab] OR "MSSQ"[tiab] OR "KSQ"[tiab] OR "Kellner's Symptom Questionnaire"[tiab] OR "DCPR"[tiab] OR "Diagnostic Criteria for Psychosomatic Research"[tiab] OR "PSI"[tiab] OR "Psychosocial Index"[tiab] OR "DAQ"[tiab] OR "Dysfunction Analysis Questionnaire"[tiab] OR "iMTA"[tiab] OR "IMCQ"[tiab] OR "iPCQ"[tiab] OR "medical consumption questionnaire"[tiab])_ AND ("English"[LA] NOT ("Animals"[MeSH] NOT "Humans"[MeSH]) NOT ("Case Reports"[ptyp] OR "case report"[ti] OR "Review"[ptyp] OR "review"[ti]))

Supplement 1b – Embase

(exp hypophysis tumor/OR "Pituitary Neoplasm*".ti,ab. OR exp hyperprolactinemia/OR "Hyperprolactinemia".ti,ab. OR "Hyperprolactinaemia".ti,ab. OR "pituitary adenoma*".ti,ab. OR exp prolactinoma/OR "Prolactinoma*".ti,ab. OR "Microprolactinoma*".ti,ab. OR "Macroprolactinoma*".ti,ab. OR "Giant prolactinoma*".ti,ab. OR "Pituitary Tumor*".ti,ab. OR exp hyperpituitarism/OR exp acromegaly/OR "Acromegal*".ti,ab. OR exp gigantism/OR "Gigantism".ti,ab. OR exp growth hormone secreting adenoma/OR "growth hormone-secreting pituitary adenoma".ti,ab. OR "growth hormone secreting pituitary adenoma".ti,ab. OR exp Cushing disease/OR "Cushing disease".ti,ab. OR "pituitary acth hypersecretion".ti,ab. OR "ACTH-Secreting Pituitary Adenoma*".ti,ab. OR "Corticotroph Adenoma*".ti,ab. OR exp Cushing syndrome/OR "Cushing syndrome".ti,ab. OR "Cushing's Syndrome".ti,ab. OR "Hypercortisolism".ti,ab. OR "Cushing's disease".ti,ab. OR "non-functioning adenoma*".ti,ab. OR "non-functioning pituitary adenoma*".ti,ab. OR "non-functioning macroadenoma*".ti,ab. OR "nonfunctioning adenoma*".ti,ab. OR "nonfunctioning pituitary adenoma*".ti,ab. OR "nonfunctioning pituitary macroadenoma*".ti,ab. OR "nonfunctioning macroadenoma*".ti,ab. OR "nonfunctioning microadenoma*".ti,ab.) AND (exp Health Care Survey/OR exp outcome assessment/OR "patient outcome assessment*" .ti,ab. OR exp Quality of Life/OR "Patient Reported Outcome Measure*".ti,ab. OR "Patient Reported Outcome*".ti,ab. OR "Patient-Reported Outcome*".ti,ab. OR "Patient Outcome Assessment*".ti,ab. OR "Patient-Centered Outcome*".ti,ab. OR "Survey*".ti,ab. OR "Questionnaire*".ti,ab. OR "patient-reported symptom*".ti,ab. OR "Life Qualit*".ti,ab. OR "Health-Related Quality Of Life".ti,ab. OR "Health Related Quality Of Life".ti,ab. OR "HR-QOL".ti,ab. OR exp patient satisfaction/OR "Patient satisfaction".ti,ab. OR "patient-reported experience measure".ti,ab. OR "Patient experience".ti,ab. OR "Health Care Survey*".ti,ab. OR "Healthcare Survey*".ti,ab. OR exp functional status/OR "Functional status".ti,ab. OR exp health Behavior/OR "Health behavior*".ti,ab. OR "Health-Related Behavior*".ti,ab. OR "Health Related Behavior".ti,ab. OR "Short-Form Health Survey*".ti,ab. _OR "Self report*".ti,ab. OR "Self-report*".ti,ab. OR "Self-reported".ti,ab. OR "Outcome instrument*".ti,ab. OR "Health scor*".ti,ab. OR "Health status".ti,ab. OR "Health outcome*".ti,ab. OR "Observer-reported".ti,ab. OR "Nurse-reported".ti,ab. OR "Caregiver-reported".ti,ab. OR "Partner-reported".ti,ab. OR "Subjective outcome*".ti,ab._ OR _"SF36".ti,ab. OR "SF-36".ti,ab. OR "SF 36".ti,ab. OR "EQ5D".ti,ab. OR "EQ-5D".ti,ab. OR "EQ 5D".ti,ab. OR "EORTC".ti,ab. OR "NHP".ti,ab. OR "Nottingham health profile".ti,ab. OR "LBNQ".ti,ab. OR "Subjective wellbeing".ti,ab. OR "Subjective well-being".ti,ab. OR "sf-20".ti,ab. OR "sf-6D".ti,ab. OR "ghq-12".ti,ab. OR "ghq-28".ti,ab. OR "ghq-30".ti,ab. OR "general health questionnaire".ti,ab. OR "gwbs".ti,ab. OR "general well-being scale".ti,ab. OR "whoqol bref".ti,ab. OR "who-qol".ti,ab. OR "World Health Organization Quality of Life Scale".ti,ab. OR "sip".ti,ab. OR "15D".ti,ab. OR "SCL-90 (-R)".ti,ab. OR "Symptom Checklist 90 (revised)".ti,ab. OR "SRT".ti,ab. OR "symptom rating test".ti,ab. OR "ACROQoL".ti,ab. OR "SSS".ti,ab. OR "Quality of Life Questionnaire".ti,ab. OR "PASQ".ti,ab. OR "Patient-assessed-Acromegaly Symptom Questionnaire".ti,ab. OR "QLS-H".ti,ab. OR "QoL-AGHDA".ti,ab. OR "HADS".ti,ab. OR "Hospital Anxiety Depression Scale".ti,ab. OR "MFI-20".ti,ab. OR "Multidimensional Fatigue Inventory".ti,ab. OR "MDI".ti,ab. OR "Major Depression Inventory".ti,ab. OR "NRS-pain".ti,ab. OR "Numerical Rating Scale-pain".ti,ab. OR "CFQ".ti,ab. OR "Cognitive Failure Questionnaire".ti,ab. OR "FACT".ti,ab. OR "Functional Assessment of Cancer Therapy".ti,ab. OR "Social Adjustment Scale".ti,ab. OR "FSFI".ti,ab. OR "Female Sexual Function Index".ti,ab. OR "SSQ".ti,ab. OR "Social Support Questionnaire".ti,ab. OR "SQ".ti,ab. OR "Symptom Questionnaire".ti,ab. OR "BDI".ti,ab. OR "Beck Depression Inventory".ti,ab. OR "MBSRQ".ti,ab. OR "Multidimensional Body-Self Relations Questionnaire".ti,ab. OR "PSLES".ti,ab. OR "Presumptive Stressful Life Events Scale".ti,ab. OR "HIT-6".ti,ab. OR "Headache Impact Test scale".ti,ab. OR "CSCL".ti,ab. OR "Coping Strategies Checklist".ti,ab. OR "AIMS2".ti,ab. OR "Arthritis Impact Measurement Scale 2".ti,ab. OR "CPRS".ti,ab. OR "Comprehensive Psychopathological Rating Scale".ti,ab. OR "MSSQ".ti,ab. OR "KSQ".ti,ab. OR "Kellner's Symptom Questionnaire".ti,ab. OR "DCPR".ti,ab. OR "Diagnostic Criteria for Psychosomatic Research".ti,ab. OR "PSI".ti,ab. OR "Psychosocial Index".ti,ab. OR "DAQ".ti,ab. OR "Dysfunction Analysis Questionnaire".ti,ab. OR "iMTA".ti,ab. OR "IMCQ".ti,ab. OR "iPCQ".ti,ab. OR "medical consumption questionnaire".ti,ab.)_ AND (English.la.) NOT ("Case Report"/OR "case report".ti,ab) NOT (exp "Review"/OR "review".ti,ab.) NOT ("rct".ti,ab.) NOT (exp "Animals"/NOT exp "Humans"/)

Supplement 1c – Web of Science

TS=("Pituitary Neoplasm*" OR "Hyperprolactinemia " OR "Hyperprolactinaemia" OR "pituitary adenoma*" OR "Prolactinoma*" OR "Microprolactinoma*" OR "Macroprolactinoma*" OR "Giant prolactinoma*" OR "Pituitary Tumor*" OR "hyperpituitarism" OR "acromegal*" OR "gigantism" OR "growth hormone-secreting pituitary adenoma" OR "growth hormone secreting pituitary adenoma" OR "pituitary acth hypersecretion" "ACTH-Secreting Pituitary Adenoma*" OR "Corticotroph Adenoma*" OR "Cushing syndrome" OR "Cushing’s Syndrome" OR "Hypercortisolism" OR "Cushing disease" OR "Cushing’s disease" OR "non-functioning adenoma*" OR "non-functioning pituitary adenoma*" OR "non-functioning macroadenoma*" OR "nonfunctioning adenoma*" OR "nonfunctioning pituitary adenoma*" OR "nonfunctioning pituitary macroadenoma*" OR "nonfunctioning macroadenoma*" OR "nonfunctioning microadenoma*") AND TS=("Health Care Survey*" OR "Patient Outcome Assessment*" OR "Quality of Life" OR "Life Qualit*" OR "Health-Related Quality Of Life" OR "Health Related Quality Of Life" OR "HR-QOL" OR "Patient Reported Outcome Measure*" OR "Patient Reported Outcome*" OR "Patient-Reported Outcome*" OR "Patient Outcome Assessment*" OR "Patient-Centered Outcome*" OR "Survey*" OR "Questionnaire*" OR "patient-reported symptom*" OR "Patient satisfaction" OR "patient-reported experience measure" OR "Patient experience" OR "Health Care Survey*" OR "Healthcare Survey*" OR "Functional Status" OR "Functional Status" OR "Health Behavior" OR "Health Behavior" OR "Short-Form Health Survey*" OR "Functional status" OR "Health behavior*" OR "Health-Related Behavior*" OR "Health Related Behavior" OR "Self report*" OR "Self-report*" OR "Self-reported" OR "Outcome instrument*" OR "Health scor*" OR "Health status" OR "Health outcome*" OR "Observer-reported" OR "Nurse-reported" OR "Caregiver-reported" OR "Caregiver-reported" OR "Partner-reported" OR "Subjective outcome*" OR "SF36" OR "SF-36" OR "SF 36" OR "EQ5D" OR "EQ-5D" OR "EQ 5D" OR "EORTC" OR "NHP" OR "Nottingham health profile" OR "LBNQ" OR "Subjective wellbeing" OR "Subjective well-being" OR "sf-20" OR "sf-6D" OR "ghq-12" OR "ghq-28" OR "ghq-30" OR "general health questionnaire" OR "gwbs" OR "general well-being scale" OR "whoqol-bref" OR "who-qol" OR "World Health Organization Quality of Life Scale" OR "sip" OR "15D" OR "SCL-90 (-R)" OR "Symptom Checklist 90 (revised)" OR "SRT" OR "symptom rating test" OR "ACROQoL" OR "SSS" OR "Quality of Life Questionnaire" OR "SSS" OR "PASQ" OR "Patient-assessed-Acromegaly Symptom Questionnaire" OR "QLS-H" OR "QoL-AGHDA" OR "HADS" OR "Hospital Anxiety Depression Scale" OR "MFI-20" OR "Multidimensional Fatigue Inventory" OR "MDI" OR "Major Depression Inventory" OR "NRS-pain" OR "Numerical Rating Scale-pain" OR "CFQ" OR "Cognitive Failure Questionnaire" OR "FACT" OR "Functional Assessment of Cancer Therapy" OR "Social Adjustment Scale" OR "FSFI" OR "Female Sexual Function Index" OR "SSQ" OR "Social Support Questionnaire" OR "SQ" OR "Symptom Questionnaire" OR "BDI" OR "Beck Depression Inventory" OR "MBSRQ" OR "Multidimensional Body-Self Relations Questionnaire" OR "PSLES" OR "Presumptive Stressful Life Events Scale" OR "HIT-6" OR "Headache Impact Test scale" OR "CSCL" OR "Coping Strategies Checklist" OR "AIMS2" OR "Arthritis Impact Measurement Scale 2" OR "CPRS" OR "Comprehensive Psychopathological Rating Scale" OR "MSSQ" OR "KSQ" OR "Kellner’s Symptom Questionnaire" OR "DCPR" OR "Diagnostic Criteria for Psychosomatic Research" OR "PSI" OR "Psychosocial Index" OR "DAQ" OR "Dysfunction Analysis Questionnaire" OR "iMTA" OR "IMCQ" OR "iPCQ" OR "medical consumption questionnaire") LA=(English) NOT TS=("veterinary" OR "rabbit*" OR "animal" OR "mouse" OR "mice" OR "rodent*" OR "rat*" OR "pig*" OR "porcine" OR "horse" OR "equine" OR "cow*" OR "bovine" OR "goat*" OR "sheep" OR "ovine" OR "canine" OR "dog*" OR "feline" OR "cat*") NOT TS=("Case Report") NOT TS=("review")

Search strategies used for (a) Pubmed, (b) Embase (c) Web of Science on September 16th, 2022.

Supplement 2 – In- and exclusion criteria

Inclusion criteria	Exclusion criteria
Population: patients with refractory hormone producing pituitary adenomas	Primarily including children
Use of patient reported outcome measures	Less than 5 refractory patients
English language	Not (yet) publicized
	Reviews, letters to editors, expert opinions, case reports
	No full text available

In- and exclusion criteria for study enrollment.

Supplement 3 – Data extraction

Data extracted	Data presented as
Study design	Cohort/cross-sectional
Number of participants	N
Number of refractory participants	N (%)
Population	acromegaly/CD/gonadotropinoma/NFPA/prolactinoma/TSH-oma/control
Female gender	N (%)
Size of adenoma: macroadenoma	N (%)
Hypopituitarism	N (%)
Treatment modality	Surgery/reoperation/TSA/craniotomy/RT/LINAC RT/med/Lanreotide/SMS/DA/CBG/Bilat. Adrenalectomy/GKS
PROM(s)	ACCQ/AcroQoL/CushingQoL/Tuebingen CD-25/PIT QOL/ BDI/EPQ-RK/EQ-5D/EQ-5D-5L/ HADS/MBSRQ/ SCL-90-R/SF-12/SF-36/RAND-26/TPQ/15D
Results of PROMs	Mean ± SD or median [IQR] unless specified otherwise
Duration of disease	Month/year
Duration of follow-up	Week/month/year

Data extracted from included articles. *AcroQoL* Acromegaly Quality of Life Questionnaire, *ACCQ* Acromegaly Comorbidities & Complaints Questionnaire, *bilat.* bilateral, *BDI* Beck Depression Inventory, *CBG* cabergoline, *CushingQoL* Cushing Quality of Life Questionnaire, *CD* Cushing's Disease, *DA* dopamine agonist, *EQ-5D* EuroQoL-5, *EQ-5D-5L* 5-level EuroQoL-5, *GKS* gamma knife surgery, *HADS* Hospital Anxiety and Depression Scale, *IQR* interquartile range, *LINAC* linear accelerator, *MBSRQ* multidimensional body-self relations questionnaire, *med* medication, *NFPA* non-functioning pituitary adenoma, *PIT QOL* Pituitary Quality of Life, *PROM* patient reported outcome measure, *SCL-90-R* Symptom Checklist-90-Revised, *SD* standard deviation, *SF-36* Short Form 36, *RAND-26* Research and Development-36, *RT* radiotherapy, *TSA* transsphenoidal adenectomy, *TSH-oma* thyroid stimulating hormone producing pituitary adenoma, *Tuebingen CD-25* Tuebingen Cushing's disease Quality of Life Inventory, *15D* 15-dimentional.

Supplement 4 - description of used PROMs

Category	PROM	Outcomes	Results (range)	Validaty in pituitary disease	Interpretation *Higher scores indicate*
Disease specific	ACCQ [1]	Type and severity of comorbidity and complaints related to acromegaly	**Total (0-24)**	-	More discomfort
	AcroQoL [2]	HR-QoL in acromegaly	Physical (0-100)<br>Psychological (0-100)<br>Appearance[a] (0-100)<br>Personal relations[a] (0-100)<br>**Total (0-100)**	AC	Better HR-QoL
	CushingQoL [3]	HR-QoL in Cushing's Disease	Psychosocial (0-100)<br>Physical (0-100)<br>**Total: (0-100)**	CD	Better HR-QoL
	Tuebingen CD-25 [4, 5]	HR-QoL in Cushing's Disease	Depression (0-100)<br>Sexual activity (0-100)<br>Environment (0-100)<br>Eating behavior (0-100)<br>Bodily restrictions (0-100)<br>Cognition (0-100)<br>**Total (0-100)[b]**	CD	Worse HR-QoL
Pituitary specific	PIT QOL [6]	HR-QoL in patients with pituitary disease	General and emotional (0-126)<br>Social (0-56)<br>Health problems related to pituitary disease (0-140)<br>Treatment related (0-21)<br>Relationship with physician (0-28)<br>**Total (0-371)**	-	Better HR-QoL
Generic HR-QoL	EQ-5D-5L [8, 9]	General HR-QoL	Mobility (0-5)<br>Self-care (0-5)<br>Usual activities (0-5)<br>Pain/discomfort (0-5)<br>Anxiety/depression (0-5)<br>**EQ-5D index scores (0-1)**<br>**VAS (0-100)**	-	Worse HR-QoL
	SF12/SF-36 /RAND-36 [11-13]	General HR-QoL	Physical functioning (0-100)<br>Role physical (0-100)<br>Bodily pain (0-100)<br>General Health (0-100)<br>Social functioning (0-100)<br>Role emotional (0-100)<br>Mental health (0-100)<br>Vitality (0-100)<br>**Mental component score (0-100)**<br>**Physical component score (0-100)**	-	Better HR-QoL

	15D [14]	General HR-QoL	Moving (0-1)<br>Seeing (0-1)<br>Hearing (0-1)<br>Breathing (0-1)<br>Sleeping (0-1)<br>Eating (0-1)<br>Speech (0-1)<br>Eliminating (0-1)<br>Usual activities (0-1)<br>Mental functioning (0-1)<br>Discomfort (0-1)<br>Depression (0-1)<br>Distress (0-1)<br>Vitality (0-1)<br>Sexual function (0-1)<br>**Total (0-1)**	-	Better HR-QoL
Symptom specific	TPQ [15, 16]	Habitual behavior	Novelty seeking (0-34)<br>Harm avoidance (0-34)<br>Reward dependence (0-30)	-	Stronger emphasis on habitual behavior
	BDI [18]	Signs and intensity of depression	**Total (0-21)**[c]	-	Worse depression
	HADS [21]	Depression and anxiety in hospital or outpatient clinic settings	**Total (0-21)**	-	More anxiety and depression
	SCL-90-R [22]	Psychopathology	Somatization (0-100)<br>Obsessiveness-compulsiveness (0-100)<br>Interpersonal sensitivity (0-100)<br>Depression (0-100)<br>Anxiety (0-100)<br>Hostility (0-100)<br>Phobic anxiety (0-100)<br>Paranoid ideation (0-100)<br>Psychoticism (0-100)<br>**Global severity Index (0-100)**<br>**Positive Symptom Distress Index (0-100)**<br>**Positive Symptom Total (0-100)**	-	Higher distress or disturbance
	MBSRQ [25, 26]	Body satisfaction	Appearance evaluation (1-5)<br>Appearance orientation (1-5)<br>Fitness evaluation (1-5)<br>Fitness orientation (1-5)<br>Health evaluation (1-5)<br>Health orientation (1-5)<br>Body areas satisfaction (1-5)<br>**Mean item score** (1-5)	-	More body image satisfaction

AcroQoL Acromegaly Quality of Life Questionnaire, *ACCQ* Acromegaly Comorbidities & Complaints Questionnaire, *BDI* Beck Depression Inventory, *CushingQoL* Cushing Quality of Life Questionnaire, *EQ-5D-5L* 5-level EuroQoL-5, *GHD* growth hormone deficiency, *HADS* Hospital Anxiety and Depression Scale, *MBSRQ* multidimensional body-self relations questionnaire, *NA* not applicable, *NHP* Nottingham Health Profile, *PIT QOL*

Pituitary Quality of Life, *PROM* patient reported outcome measure, *SCL-90-R* Symptom Checklist-90-Revised, *SF-36* Short Form 36, *RAND-26* Research and Development-36, *TPQ* Cloninger's Tridimensional Personality Questionnaire, *Tuebingen CD-25* Tuebingen Cushing's Disease Quality of Life Inventory, *15D* 15-Dimentional.

[a] Psychological subscale

[b] Nader et al. [28] presented categorized results (mild/severe). Mild: scores > percentile rank 84 of age- and gender-specific cut-off values. Severe: scores > percentile rank 95 of age- and gender-specific cut-off values.

[c] Alcalar et al.[29] and Nader et al. [28] presented categorized results using different cutoff values. Alcalar et al.: <17 points: absence of depression. ≥17 points: presence of depression. Nader et al.: ≤10 points: no depression, 11-17 points: mild to moderate depression, ≥18 points: severe depression.

[d] The 34-item version was used

[e] more negative scores also indicate bigger discrepancy between perceived and ideal body type

Supplement 5 - ISOQOL scoring details

Section	Criterium	Scoring details
Title and abstract	The PRO should be identified as an outcome in the abstract	
	For 1° outcome: The title of the paper should be explicit as to the cohort study including a PRO	This item was scored 1 if at least quality of life, wellbeing, symptoms or related terms were mentioned in the title
Introduction, background and objectives	The PRO hypothesis should be stated and should specify the relevant PRO domain(s) if applicable	
	For 1° outcome: The introduction should contain a summary of PRO research that is relevant to the cohort study	
	For 1° outcome: Additional details regarding the hypothesis should be provided, including the rationale for the selected domain(s), the expected direction(s) of change, and the time points for assessment	
Outcomes registration	The mode of administration of the PRO tool and the methods of collecting data (e.g. telephone, other) should be described	
	The rationale for choice of the PRO instrument used should be provided	This item was also scored 1 if the used PROM was disease-specific
	Evidence of PRO instrument validity and reliability should be provided or cited	
	The intended HRQL data collection schedule should be provided	This item was scored NA in cross-sectional studies
	PROs should be identified in the trial protocol; post hoc analyses should be identified	This item was scored NA if there was no need for post hoc testing because only two groups were compared
	The status of PRO as either a primary or secondary outcome should be stated	This item was also scored 1 if the PROM was the only outcomes and therefore clearly the primary outcome
	For 1° outcome: A citation for the original development of the PRO instrument should be provided	
	For 1° outcome: Windows for valid PRO responses should be specified and justified as being appropriate for the clinical context	
Sample size	For 1° outcome: There should be a power/sample size calculation relevant to the PRO based on a clinical rationale (e.g. anticipated effect size)	
Statistical methods	There should be evidence of appropriate statistical analysis and tests of statistical significance for each PRO hypothesis tested	
	Statistical approaches for missing data should be explicitly stated, and the extent of missing data should be stated	This item was scored 1 if explicitly stated that there was no missing data
	For 1° outcome: The manner in which multiple comparisons have been addressed should be provided	
Participant flow	A flow diagram or a description of the allocation of participants (if applicable) and those lost to follow-up should be provided for PROs specifically	This item was scored NA in cross-sectional non-intervention studies
	The reasons for missing data should be explained	
Baseline data	The study patients' characteristics should be described, including baseline PRO scores	
Outcomes and estimation	The analysis of PRO data should account for survival differences between treatment groups if relevant	This item was scored NA if survival was not an outcome
	Results should be reported for all PRO domains (if multi-dimensional) and items identified by the reference instrument (i.e. not just those that are statistically significant)	
	The proportion of patients achieving predefined responder definitions should be provided where relevant	This item was scored NA if there was no PROM-based responder definition
Limitations	The limitations of the PRO components of the study should be explicitly discussed	
Generalizability	Generalizability issues uniquely related to the PRO results should be discussed, if applicable	
Interpretation	The clinical significance of the PRO findings should be discussed	This item was only scored 1 in studies that explicitly described the meaning and importance of the PRO findings in clinical context
	The PRO results should be discussed in the context of the other clinical studies	

Protocol	A copy of the instrument should be included if it has not been published previously (1 if published previously)	
Percentage of items reported by study (%)		The percentage was calculated as total points divided by the number of applicable items, multiplied by 100%

International Society for Quality of Life Research (ISOQOL) criteria modified for non-randomized controlled trials with details about scoring of this review. *1° outcome* primary outcome, *NA* not applicable, *PRO* patient reported outcome, *PROM* patient reported outcome measure.

Supplement 6 – Flowchart of article screening and enrollment

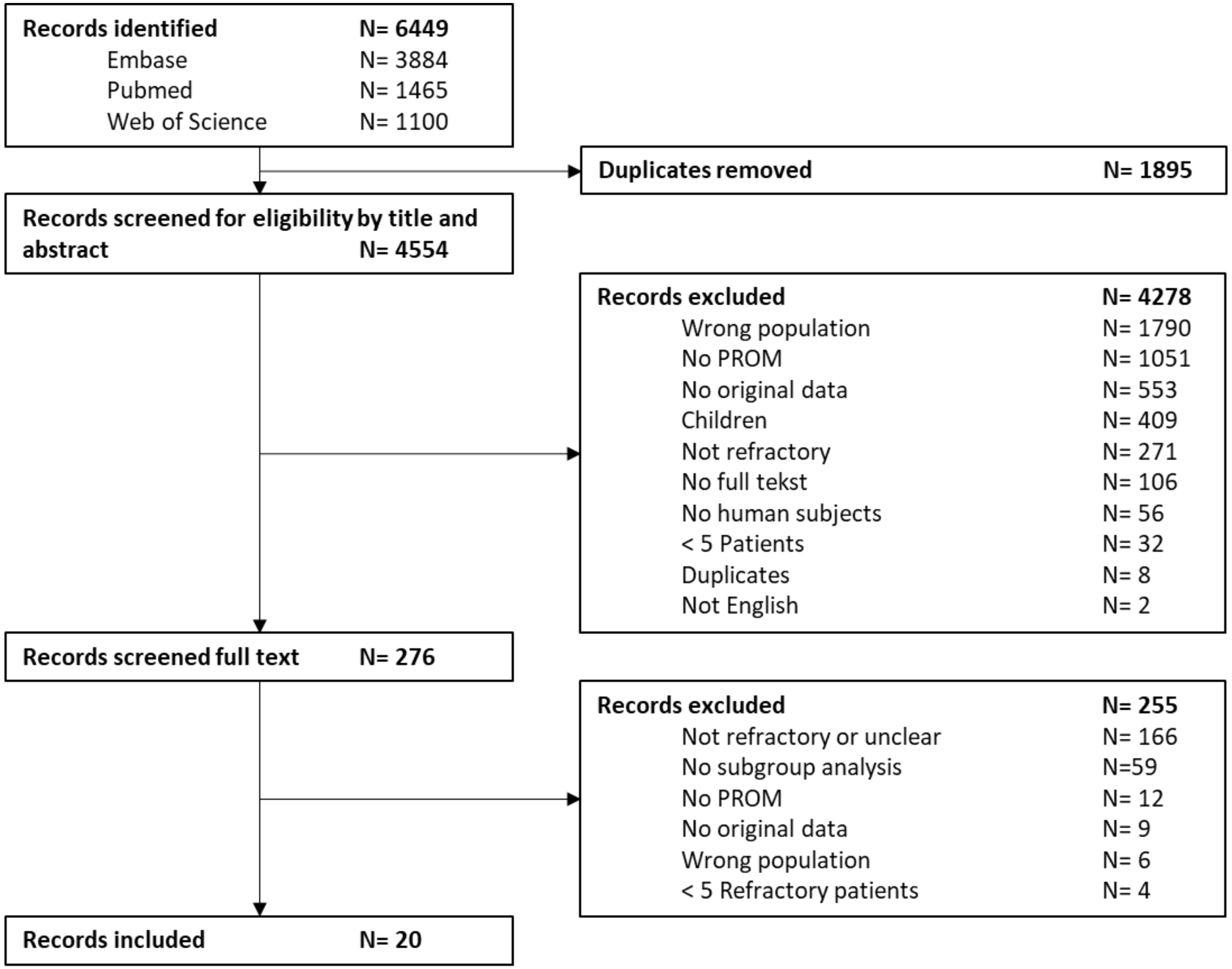

Flowchart of article screening and enrollment that was used in PubMed, Embase and Web of Science (searched on September 16th, 2022). *PROM* patient reported outcome measure.

Supplement 7 – Study characteristics per study

First author, Study design	Population	Subgroup	N	Treatment	Age in years	Sex (female)	Macroadenoma	Hypopituitarism	Duration disease	Duration follow-up
**Alcalar [29]**<br><br>Cross-sectional study	CD, Healthy controls	Total	Total: 80<br>CD: 40<br>Control: 40	Primary:<br>Surgery: 40 (100.0%)<br>Additional:<br>None: 29 (72.5%)<br>Bilat. adrenalectomy[a]: 4 (10.0%)<br>GKS: 3 (7.5%)<br>Bilat adrenalectomy + GKS: 4 (10.0%)	Total: NR<br>CD: 39.6 ± 10.6<br>Control: 35.66 ± 9.1	Total: 55 (68.8%)<br>CD: 31 (77.5%)<br>Control: 24 (60.0%)	10 (25.0%)	Any: NR<br>TSH: 7 (17.5%)<br>LH/FSH: 3 (7.5%)<br>DI: 1 (2.5%)<br>ACTH: 4 (10.0%)	NR	NA
		Refractory	8 (20.0%)	NR	NR	NR	NR	NR	NR	NA
**Carluccio [27]**<br><br>Cross-sectional study	CD	Total	102	TSA: 102 (100.0%) [b]	43.1 (range 14-73)	78 (76.5%)	22 (21.6%)	34 (33.3%)	NR	NA
		Refractory	8 (7.8%)	NR	NR	NR	NR	NR	NR	NA
**Chin [30]**<br><br>Cohort study	AC	Total	58	Current:<br>Lanreotide: 58 (100.0%)<br>Previous:<br>Surgery only: 40 (69.0%)<br>GKS only: 1 (1.7%)<br>Surgery + GKS +/- RT 12: (20.7%)[c]<br>None: 5 (8.6%)	47 (range 21-72)	29 (50.0%)	NR	0 (0.0%)	NR	24 w
		Refractory	36 (62.1%)	NR	NR	NR	NR	0 (0.0%)	NR	NR
**Dantas [31]**<br><br>Cross-sectional	AC	Total	42	Surgery: 32 (76.2%)<br>Two surgeries: 10 (23.6%)<br>Surgery + RT: 14 (34.4%)<br>Primary med: 10 (23.8%)	49.6, 95% CI: 45.6-53.7	22 (52.4%)	Not reported correctly[d]	NR	12.74 y, CI 95%: 11.64-15.83	NA
		Refractory	14 (33.3%)[e]	NR	NR	NR	NR	NR	NR	NA

[a] Of which adenomectomy 27 (67.5%), hemihypophysectomy 8 (20.0%), adenomectomy + hemihypophysectomy 3 (7.5%), craniotomy 2 (5.0%).
[b] surgery + GKS: 9 (15.5%), surgery + GKS + CRT: 3 (5.2%).
[c] Age at diagnosis.
[d] Percentages of micro- and macroadenomas in men add up to 113%.
[e] Number of patients not reported in article. Data was shared by author upon request.

First author, Study design	Population	Subgroup	N	Treatment	Age in years	Sex (female)	Macroadenoma	Hypopituitarism	Duration disease	Duration follow-up
**Dimopoulou [32]**<br><br>Cross-sectional Study	CD, NFPA, Healthy controls	Total	Total: 210<br>CD: 50<br>NFPA: 60<br>Control: 100	Total: NR<br><br>CD:<br>Surgery: 49 (98.0%)<br>RT: 13 (26.0%)<br>Med: 5 (10.0%)<br><br>NFPA:<br>Surgery: 52 (86.7%)<br>RT: 15 (25.0%)<br>Med: 0 (0.0%)<br><br>Control:<br>NA	Total: NR<br>CD: 46.4 ± 11.6<br>NFPA: 60 ± 10.6<br>Control: 46.4 ± 11.6	Total: 144 (68.6%)<br>CD: 41 (82.0%)<br>NFPA: 21 (35.0%)<br>Control: 82 (82.0%)	Total[f]: 60 (54.5%)<br>CD: 10 (20.0%)<br>NFPA: 50 (83.3%)	Total any[f]: 84 (70.0%)<br>CD any: 32 (64.0%)<br>NFPA any: 45 (75.0%)	NR	NA
		Refractory	CD: 13 (26.0%)	NR	NR	NR	NR	NR	NR	NA
**Fathalla [33]**<br><br>Cross-sectional study	AC, incidentalomas	Total	20	Surgery: 20 (100.0%)<br>Med: 7 (35.0%)<br>RT: 1 (5.0%)<br>Reoperation: 5 (25.0%)	42 ± 13.5	11 (55.0%)	NR[g]	4 (20.0%)	NR[h]	NA
		Refractory	6 (30.0%)	NR	NR	NR	NR	NR	NR	NA
**Gu [34]**<br><br>Cohort study	AC	Total	154	TSA: 154 (100.0%)<br>SMS before surgery: 29 (19.2%)	43.9 ± 12.3	76 (50.3%)	NR	NR	NR	6 mos
		Refractory	44 (28.7%)	NR	43.6 (12.9%)	20 (45.5%)	NR	NR	NR	NR
**Guo [35]**<br><br>Cross-sectional	AC	Total	327	Surgery 265 (81.0%)[i]<br>Med: 160 (48.9%)[j]<br>RT: 110 (33.6%)	39.2 ± 9.5	201 (61.5%)	NR	NR	NR[k]	NA
		Refractory	154 (47.1%)	NR	NR	NR	NR	NR	NR	NA

[f] Percentage of NFPA + CD (N=110).
[g] Median tumor volume 3.8 [IQR 1.4-6.2].
[h] Follow-up time 11 months ± 3.1 months.
[i] Endoscopic TSA 131 (40.1%), microscopic TSA 122 (37.3%), craniotomy 12 (3.7%).
[j] SMS 139 (42.5%), DA 70 (21.4%), SMS+DA 49 (15.0%).
[k] Mean time from initial treatment to surveys was 10 ± 6.2 years.

First author, Study design	Population	Subgroup	N	Treatment	Age in years	Sex (female)	Macroadenoma	Hypopituitarism	Duration disease	Duration follow-up
**Hua [36]**<br><br>Cross-sectional	AC	Total	52	Total: NR<br><br>Controlled:<br>Surgery: 28 (93.3%)<br>Reoperation: 10 (33.3%)<br>Lanreotide: 13 (43.3%)<br>RT: NR[l] (64.0%)	51.9 ± 10.1	25 (50.0%)	35 (67.3%)	Any: 16 (30.8%)<br>TSH: 11 (2.1%)<br>ACTH: 15 (2.9%)<br>FSH/LH: 6 (11.5%)[m]	12.6 ±7.1 y	NA
		Refractory	Total: 22 (42.3%)<br><br>SMS (+): 11 (21.2%)<br><br>SMS (-): 11 (21.2%)	Total:<br>Surgery: 16 (72.7%)<br>Reoperation: 0 (0.0%)<br>Lanreotide: 11 (50.0%)<br>RT: 3 (13.6%)<br><br>SMS(+):<br>Surgery: 9 (81.8%)<br>Reoperation: 0 (0.0%)<br>RT: 2 (18.2%)<br><br>SMS (-):<br>Surgery: 7 (63.6%)<br>Reoperation: 0 (0.0%)<br>RT: 1 (9.1%)	Total:<br>52.0 ± 12.1<br><br>SMS (+):<br>48.9 ± 12.7<br><br>SMS (-):<br>55.0 ± 11.1	Total:<br>8 (36.4%)<br><br>SMS (+):<br>5 (45.5%)<br><br>SMS (-):<br>3 (27.3%)	Total:<br>NR[n] (75.0%)<br><br>SMS (+):<br>10 (90.9%)<br><br>SMS (-):<br>6 (54.5%)	Total:<br>4 (18.2%)<br><br>SMS (+):<br>3 (27.3%)<br><br>SMS (-):<br>1 (9.1%)	Total:<br>10.0 ±7.3<br><br>SMS(+):<br>11.1 ± 8.2<br><br>SMS (-):<br>8.8 ± 6.4	NA
**Milian [38]**<br><br>Cohort study	AC, CD, PRL, NFPA, other[18]	Total	Total: 106<br>AC: 29<br>CD: 14<br>PRL: 12<br>NFPA: 39<br>Other: 12[o]	TSA: 106 (100.0%)<br>RT: 4 (3.8%)	48.0 ± 16.0	69 (65.1%)	NR	Preoperative:<br>Any: 35 (33.0%)<br><br>3 mos postoperative:<br>Any: NR (24.1%)[p]	NR	12 mos
		Refractory	Total:14(13.2%)<br>AC: 10 (34.5%)<br>CD[q]: 3 (21.4%)<br>PRL[q, r]: 1(14.3%)	NR	NR	NR	NR	NR	NR	NR

[l] Number of patients who received RT not reported. Percentage cannot be converted to an absolute value due to unreported missing data or a typing error.
[m] Patients on replacement therapy reported only. Unclear if all patients with hypopituitarism were on replacement therapy.
[n] Total number of refractory patients with macroadenoma not reported. Percentage reported does not correspond with the sum of SMS(+) and SMS (-) and cannot be converted to a number of patients.
[o] Rathke's cleft cyst, sellar colloid cysts.
[p] Number of patients with any hypopituitarism not reported. Percentages cannot be converted to numbers due to unreported missing data or a typing error.
[q] Refractory CD and PRL patients were not included in further analysis, as N<5.
[r] Data available of 7 patients, missing data N=5.

First author, Study design	Population	Subgroup	N	Treatment	Age in years	Sex (female)	Macroadenoma	Hypopituitarism	Duration disease	Duration follow-up
**Nader [28]**<br><br>Cross-sectional study	CD	Total	54	Primary:<br>TSA: 54 (100.0%)<br>RT: 3 (5.6%)<br>Bilat. adrenalectomy: 4 (74.0%)<br>Reoperation Nelson's Tumor: 1 (1.9%)	48.0 ± 15.5	41 (75.9%)	5 (9.3%)	NR	NR[s]	NA
		Refractory	8 (14.8%)	NR	NR	NR	NR	NR	NR	NA
**Psaras [1]**[t]<br><br>Cross-sectional study	AC	Total	55	Primary:<br>Surgery 55 (100.0%)<br>Additional:<br>Reoperation: 8 (14.5%)<br>RT: 5 (9.1%)<br>Med: 15 (27.3%)	54.1 ± 15.1	28 (50.9%)	48 (87.3%)	NR	NR	NA
		Refractory	18 (32.7%)	NR	NR	NR	NR	NR	NR	NA
**Psaras [39]**[t]<br><br>Cross-sectional study	AC, Healthy controls	Total	Total: 89<br>AC: 37<br>CD: 24<br>Control: 28	Total any surgery: 61 (100.0%)[u]<br>AC TSA: 36 (97.3%)<br>CD TSA: 24 (100.0%)	Total: NR<br>AC: 52.1 ± 14.7<br>CD: 52.6 ± 15.7<br>Control: 48.9 ± 2 1.3	Total: 54 (55.1%)<br>AC: 18 (48.6%)<br>CD: 17 (70.8%)<br>Control: 19 (67.9%)	Total: 36 (59.0%)[u]<br>AC: 32 (86.5%)<br>CD: 4 (16.7%)	Total any: 16 (26.2%)[u]<br>CD any: 13 (54.2%)[v]<br>AC any: 3 (8.1%)	NR	NA
		Refractory	Total: 19 (21.3%)<br>AC: 14 (37.8%)<br>CD: 5 (20.8%)	NR	NR	NR	NR	NR	NR	NA

[s] Average time between surgery and completion of questionnaires is 3 years (range 1 - 6 years).
[t] Psaras et al. [1] and Psaras et al. [39] report on overlapping populations.
[u] Percentage of AC + CD patients.
[v] All 23 CD patients received replacement therapy because of hypocortisolism.

First author, Study design	Population	Subgroup	N	Treatment	Age in years	Sex (female)	Macroadenoma	Hypopituitarism	Duration disease	Duration follow-up
**Raappana [40]**<br><br>Cohort study	AC, CD, PRL, NFPA	Total	Total: 98<br>AC: 22<br>CD: 6<br>PRL: 17<br>NFPA: 53	Total:<br>TSA: 92 (93.9%)<br>Craniotomy: 12 (12.2%)<br>DA treatment: 7 (7.1%)<br>RT: 14 (14.3%)<br><br>AC:<br>Reoperation: 7 (31.8%)<br>Craniotomy: 4 (18.2%)<br>Med: 9 (40.9%)<br>RT: 6 (27.2%)<br><br>CD:<br>Reoperation: 2 (33.3%)<br>Craniotomy: 0 (0.0%)<br>Med:0 (0.0%)<br>RT: 0 (0.0%)<br><br>PRL:<br>Reoperation: 1 (5.9%)<br>Craniotomy: 4 (23.5%)<br>med: 7 (41.2%)<br>RT: 2 (11.8%)<br><br>NFPA:<br>Reoperation: 12 (22.6%)<br>Craniotomy: 4 (1.9%)<br>Med: 0 (0.0%)<br>RT: 6 (11.3%)	Total:<br>mean 52.8 (95% CI: 49.6-56)<br><br>AC:<br>Mean 45.0 (95% CI: 39.0-51.0)<br><br>CD:<br>Mean 34.8 (95% CI: 20.0-50.0)<br><br>PRL:<br>Mean 46.4 (95% CI: 40.4-52.4)<br><br>NFPA:<br>Mean 60.0 (95% CI: 56.2-64.2)	Total: 53 (54.1%)<br>AC: 10 (45.5%)<br>CD: 5 (83.3%)<br>PRL: 12 (70.5%)<br>NFPA: 26 (48.1%)	Total: 82 (83.7%)<br>AC: 16 (72.7%)<br>CD: 2 (33.3%)<br>PRL: 11 (64.7%)<br>NFPA: 53 (100%)	Total any: 50 (51.0%)<br>AC any: 9 (40.9%)<br>CD any: 1 (16.7%)<br>PRL: 8 (47.1%)<br>NFPA: 32 (60.4%)	NR	Total: mean 6.3 y (95% CI: 5.4-7.1)<br><br>CD: mean 6.0 y (95% CI: 1.1-10.8)<br><br>AC: mean 7.8 y (95% CI: 6.1-9.5)<br><br>PRL: mean 9.4 y (95% CI: 7.5-11.)<br><br>NFPA: mean 4.7 y (95% CI: 3.6-5.7)
		Refractory	Total: 13 (13.3%)<br>AC[w]: 3 (13.6%)<br>CD[w]: 1 (16.7%)<br>PRL: 5 (29.4%)	NR	NR	NR	NR	NR	NR	NR

[w] Refractory AC and CD patients were not included in further analysis as N<5.

First author, Study design	Population	subgroup	N	Treatment	Age in years	Sex (female)	Macroadenoma	Hypopituitarism	Duration Disease	Duration follow-up
**Ritvonen [41]**<br><br>Cross-sectional	AC, CD, PRL, TSH, GON	Total	Total: 100<br>AC: 47<br>CD: 21<br>PRL: 26<br>TSH[x]: 2<br>GON: 4<br><br>Control: 4924	Total:<br>TSA 100 (100.0%)<br>Reoperation: 6 (6.0%)<br>Med: 37 (37.0%)<br>RT: 8 (8.0%)<br><br>AC:<br>TSA: 47 (100.0%)<br>Reoperation: 3 (6.4%)<br>RT: 5 (10.6%)<br>Med: 16 (34.0%)<br><br>CD:<br>TSA: 21 (100.0%)<br>Reoperation: 2 (9.5%)<br>RT: 1 (4.8%)<br>Med: 5 (23.8%)<br><br>GON:<br>TSA: 4 (100.0%)<br>Reoperation: 1 (25.0%)<br>RT: 2 (50.0%)<br>Med: 0 0.0%)<br><br>PRL:<br>TSA: 26 (100.0%)<br>Reoperation: 0 (0.0%)<br>RT: 0 (0.0%)<br>Med: 16 (61.5%)	Total: 53.1 ± 1.4<br>AC: 56.3 ± 12.5<br>CD: 52.3 ± 12.8<br>GON: 48.3 ± 17.0<br>PRL: 47.3 ± 16.7	Total: 58 (58.0%)<br>AC: 21 (44.7%)<br>CD: 18 (85.7%)<br>GON: 2 (50.0%)<br>PRL 16 (61.5%)	Total: 72 (72.0%)<br>AC: 39 (83.0%)<br>CD: 7 (33.3%)<br>GON: 3 (75.0%)<br>PRL: 21 (80.8%)	Total: 43 (43.9%)<br>AC: 21 (44.7%)<br>CD: 10 (47.6%)<br>GON: 2 (50.0%)<br>PRL: 7 (26.8%)	NR	NA
		Refractory	Total[y]: 10 (10.0%)<br>AC: 5 (10.6%)<br>CD[z]: 1 (4.8%)<br>GON: 0 (0.0%)<br>PRL[z]: 4 (15.4%)	NR	NR	NR	NR	NR	NR	NA

[x] TSH-producing adenoma not included in further analysis by author.

[y] Table 1 reports 10 patients not in hormonal remission, however in the text 9 patients are reported to have hormonally active disease.

[z] Refractory CD and PRL patients were not included in further analysis as N<5.

First author, Study design	Population	subgroup	N	Treatment	Age in years	Sex (female)	Macroadenoma	Hypopituitarism	Duration disease	Duration follow-up
**Tepp [42]**<br><br>Cross-sectional study	AC, NFPA	Total	Total: 55<br>AC: 33<br>NFPA: 22	Total:<br>TSA only: 26 (47.3%)<br>TSA + RT: 6 (10.9%)<br>Surgery + med: 2 (3.6%)<br>TSA + RT + med: 17 (30.9%)<br>Craniotomy + RT: 1 (1.8%)<br>Craniotomy + TSA + RT: 2 (3.6%)<br>RT only: 1 (1.8%)<br><br>AC:<br>TSA only: 10 (30.3%)<br>TSA + RT: 3 (9.1%)<br>surgery + med: 2 (6.1%)<br>TSA + RT + med: 17 (51.5%)<br>RT only: 1 (6.1%)<br><br>NFPA:<br>TSA only: 16 (72.7%)<br>TSA + RT: 3 (13.6%)<br>Craniotomy + RT: 1 (4.5%)<br>Craniotomy + TSA + RT: 2 (9.1%)	Total: NR<br>AC: 50.8 ± 10.7<br>NFPA: 61.5 ± 14.1	Total: 24 (43.6%)<br>AC: 14 (42.4%)<br>NFPA: 10 (45.5%)	Total: 40 (72.7%)<br>AC: 19 (57.%)<br>NFPA: 21 (95.5%)	NR	AC: 15.5 ± 11.2 y<br><br>NFPA: 6.5 ± 7.9 y	NA
		Refractory	6 (18.2%)	NR	NR	NR	NR	NR	NR	NA

First author, Study design	Population	subgroup	N	Treatment	Age in years	Sex (female)	Macroadenoma	Hypopituitarism	Duration disease	Duration follow-up
**Vandeva [43]**<br><br>1. Cross-sectional<br><br>2. Cohort study	AC	Total	**Cross-sectional:**<br>Total: 212<br>Active: 100<br>Controlled: 112<br><br>**Cohort[aa]:**<br>Total: 70<br>Controlled: 45	**Cross-sectional**:<br>Total:<br>TSA: 121 (57.1%)<br>2 or more TSA: 57 (26.9%)<br>RT: 47 (22.2)<br>Med: 105 (49.5%)<br>Active:<br>TSA: 44 (44.0),<br>2 or more TSA: 25 (25.0%)<br>RT: 17 (17.0%)<br>Med: 41 (41.0%)<br>Controlled:<br>TSA: 77 (68.8%)<br>2 or more TSA: 32 (28.6%)<br>RT: 30 (26.8%)<br>Med: 64 (57.1%)<br><br>**Cohort**:<br>Total:<br>TSA: 43 (61.4)<br>2 or more TSA: 25 (35.7%)<br>RT 19 (27.1%)<br>Med: 58 (82.9%)	**Cross-sectional**:<br>Total: NR<br>Active: 49.5 ± 12.9<br>Controlled: 52.3 ± 11.6<br><br>**Cohort**:<br>NR	**cross-sectional**:<br>Total: 134 (63.2%)<br>Active: 61 (61.0%)<br>Controlled: 73 (65.2%)<br><br>**Cohort**:<br>Total: 48 (68.6%)	NR	**Cross-sectional**:<br>Total: 77 (35.2%)<br>Active: 44 (39.3%)[bb]<br>Controlled: 33 (33.0%)[35]<br><br>**Cohort**:<br>Total: NR<br>Controlled: 12 (28.9%)	**Cross-sectional:**<br>Active: 6.9 ± 7.5 y<br>Controlled: 6.9 ± 7.8 y<br><br>**Cohort**:<br>NR	**Cross-sectional**:<br>NA<br><br>**Cohort**:<br>Total: NR<br>Controlled: 29.3 ± 18.8 mos
		Refractory	**Cross-sectional:**<br>0 (0.0%)<br><br>**Cohort**<br>25 (35.7%)	**Cross-sectional:**<br>-<br><br>**Cohort:**<br>TSA 12 (48.0%)<br>2 or more TSA: 11 (44.0%)<br>RT: 6 (24%)<br>Med: 25 (88.0%)	NR	**Cross-sectional:**<br>-<br><br>**Cohort:**<br>17 (68.0%)	NR	**Cross-sectional:**<br>-<br><br>**Cohort:**<br>7 (28.0%)	NR	**Cross-sectional:**<br>-<br><br>**Cohort:**<br>29 ± 19.7 mos

[aa] subset of patients with active disease at time of cross-section were enrolled in cohort study.
[bb] Numbers do not correspond with percentage due to not reported missing data or a typing error.

First author, Study design	Population	subgroup	N	Treatment	Age in years	Sex (female)	Macroadenoma	Hypopituitarism	Duration disease	Duration follow-up
**Vega-Beyhart [44]**<br><br>Cross-sectional study	AC, CD, PRL	Total	Total: 175<br>AC: 48<br>CD: 30<br>PRL: 53<br>NFPA: 44	Total:<br>Surgery: 76 (43.4%)<br>CBG: 102 (58.3%)<br>LINAC RT: 37 (21.1%)<br><br>AC total:<br>Surgery: 30 (62.5%)<br>CBG: 25 (52.1%)<br>LINAC RT: 20 (41.7%)<br><br>CD total:<br>Surgery: 28 (90.0%)<br>CBG: 25 (83.3%)<br>LINAC RT: 10 (33.3%)<br><br>PRL total:<br>Surgery: 6 (11.3%)<br>CBG: 49 (92.5%)<br>LINAC RT: 2 (3.8%)<br><br>NFPA total:<br>Surgery: 12 (27.2%)<br>CBG: 19 (43.2%)<br>LINAC RT: 5 (11.4%)	Total:<br>44 ± 14<br><br>AC:<br>Total: NR<br>Controlled: 36 [IQR 27-51]<br><br>CD:<br>Total: NR<br>Controlled: 29 [IQR 25-37]<br><br>PRL:<br>Total: NR<br>Controlled: 30 [IQR 25-39]<br><br>NFPA:<br>44 [IQR 36-54]	Total:<br>132 (75.4%)<br><br>AC:<br>Total: 21 (43.8%)<br><br>CD:<br>Total: 29 (95.7%)<br><br>PRL:<br>Total: 47 (88.7%)<br><br>NFPA:<br>35 (79.5%)	Total:<br>37 (21.1%)<br><br>AC:<br>Total: 15 (31.3%)<br><br>CD:<br>Total: 1 (3.3%)<br><br>PRL:<br>Total: 8 (15.1%)<br><br>NFPA:<br>13 (29.5%)	Total:<br>Pan: 25 (14.3%)<br>ACTH: 50 (28.6%)<br>GH: 15 (8.6%)<br>TSH: 19 (10.9%)<br>LH/FSH: 31 (17.8%)<br><br>AC total:<br>Pan: 7 (14.6%)<br>ACTH: 13 (27.1%)<br>GH: 1 (2.1%)<br>TSH: 4 (8.3%)<br>FSH/LH: 8 (16.7%)<br><br>CD total:<br>Pan: 4 (13.3%)<br>ACTH: 10 (33.3%)<br>GH: 2 (6.7%)<br>TSH: 3 (10.0%)<br>FSH/LH: 6 (20.0%)<br><br>PRL total:<br>Pan: 6 (11.3%)<br>ACTH: 16 (30.2%)<br>GH: 5 (9.4%)<br>TSH: 6 (11.3%)<br>FSH/LH: 10 (18.9%)<br><br>NFPA:<br>Pan: 8 (18.2%)<br>ACTH: 11 (25.0%)<br>GH: 7 (15.9%)<br>TSH: 6 (13.6%)<br>FSH/LH: 7 (15.9%)	7<br>[IQR 1-10] y	NA

		Refractory	Total: 58<br>(33.1%)<br>AC 0 (0.0%)<br>CD: 7 (23.3%)<br>PRL 28 (52.8%)	CD:<br>Surgery: 7 (100.0%)<br>CBG: 3 (42.9%)<br>LINAC RT: 3 (42.9%)<br><br>PRL:<br>Surgery: 4 (14.3%)<br>CBG 27: (96.4%)<br>LINAC RT: 1 (3.6%)	Total:<br>NR<br><br>CD:<br>27 [IQR 19-38]<br><br>PRL:<br>27 [IQR 21-34]	Total:<br>33 (94.3%)<br><br>CD:<br>7 (100.0%)<br><br>PRL:<br>26 (92.9%)	Total:<br>6 (17.1%)<br><br>CD:<br>1 (14.3%)<br><br>PRL:<br>5 (17.9%)	CD:<br>Pan: 1 (14.3%)<br>ACTH: 0 (0.0%)<br>GH: 1 (14.3%)<br>TSH: 2 (28.6%)<br>FSH/LH: 2 (28.6%)<br><br>PRL:<br>Pan: 5 (20.3%)<br>ACTH: 13 (46.4%)<br>GH: 4 (14.3%)<br>TSH: 5 (20.3%)<br>FSH/LH: 8 (28.6%)	NR	NA
**Yamamoto [45]**<br><br>Cross-sectional study	AC	Total	74	Surgery only 34 (45.9%)<br>Med after surgery: 22 (29.7%)<br>Med only: 9 (12.2%)<br>RT 9 (12.2%)<br>RT + med + surgery: 8 (10.8%)<br>RT after surgery: 1 (1.2%)	62.0 [IQR 50.7-70.0]	39 (52.7%)	NR[cc]	ACTH[41]: 7 (9.5%)<br>TSH[41]: 11 (14.9%)<br>FSH/LH[41]: 3 (4.1%)<br>GH[dd]: 1 (1.4%)	10<br>[3.0-16.0] y	NA
		Refractory	38 (51.1%)	NR	NR	NR	NR	NR	NR	NA
**Ye [46]**<br><br>Cohort study	CD, NFPA	Total	Total: 71<br>CD: 51<br>NFPA: 20	Total:<br>NR<br><br>CD:<br>TSA: 50 (98.0%)<br>Craniotomy: 1 (2.0%)	Mean: 42.4	NR	Total:<br>NR<br><br>CD [ee]:<br>10 (20.4%)	NR	NR	First:<br>mean 2.35 mos<br><br>Second:<br>mean 7.4 mos
		Refractory	CD: 7 (13.7%)	NR	Mean: 38.6	NR	NR	NR	NR	NR

General characteristics of all included studies. Characteristics are reported for the total population, subgroups and refractory patients separately if reported as such by the author. Data not presented was not reported by the author of the article. Values expressed as mean ± SD or median [interquartile range], unless specified otherwise. *AC* acromegaly, *Bilat*. bilateral, *CBG* cabergoline, *CD* Cushing's disease, *DA* dopamine agonist, *DI* diabetes insipidus, *GHRA* growth hormone receptor antagonist, *GKS* gamma knife surgery, *LINAC* linear accelerator, *mos* months, *med* medication, *NA* not applicable, *NFPA* non-functioning pituitary adenoma, *NR* not reported, *PAN* panhypopituitarism, *PRL* prolactinoma, *RCC* Rathke's cleft cyst, *RT* radiotherapy, *SMS(+)* on somatostatin analogue treatment, *SMS(-)* not on somatostatin analogue treatment, *TSA* transsphenoidal adenectomy, *w* week, *Y* years, *95% CI* 95% confidence interval.

[cc] Total median tumor size (cm): 1.3 [IQR 0.9-1.8].
[dd] Patients on replacement therapy reported only. Unclear whether all patients with hypopituitarism were on replacement therapy.
[ee] N=49, missing data: 2.

Supplement 8 – Risk of bias assessment per study

Category	Criterium	Alcalar [29]	Carluccio [27]	Chin [30]	Dantas [31]	Dimopoulou [32]	Fathalla [33]	Gu [34]	Guo [35]	Hua [36]	Milian [38]	Nader [28]	Psaras [1]	Psaras [39]	Raappana [40]	Ritvonen [41]	Trepp [42]	Vandeva [43]	Vega-Beyhart [44]	Yamamoto [45]	Ye [46]
Study population	Inclusions either consecutive patients or all eligible. Not a random sample	1	1	1	0	0	1	1	1	1	0	0	0	0	1	1	1	0	1	0	1
	All included patients with active disease showed clinical symptoms of the particular pituitary adenoma	0	0	0	1	0	0	1	0	0	0	0	0	0	0	0	0	0	0	0	0
	Description of the clinical symptoms and definitions of the symptoms	0	0	0	0	0	0	0	0	0	0	0	0	0	0	0	0	0	0	0	0
	Description of criteria for diagnosis of pituitary disease	1	1	1	1	1	0	1	1	0	0	0	0	0	1	0	0	1	1	1	0
	Criteria for diagnosis of pituitary disease according to the most recent international guidelines at the time of publication	1	1	1	1	1	0	1	1	0	0	0	0	0	1	0	0	1	0	1	0
	Definition of remission described	1	1	0	1	1	1	1	1	1	0	0	1	1	1	1	1	1	1	1	0
	Definition of remission according to most recent international guidelines at the time of publication	1	1	0	1	1	1	1	1	1	0	0	1	1	1	1	1	1	0	1	0
	Definition of refractory described	0	0	0	0	0	0	0	0	0	0	0	0	0	0	0	0	0	0	0	0
	Definition of intolerant described	0	0	0	0	0	0	0	0	0	0	0	0	0	0	0	0	0	0	0	0
	Treatment modalities of patients described	1	1	1	1	1	1	1	1	1	1	1	1	1	1	1	1	1	1	1	0
	Mention that treatment was according to most recent guidelines at time of publication	0	0	0	0	1	0	0	1	0	0	0	0	0	0	0	0	0	0	0	
Data collection	Lost to follow-up <10%<br>N (%)	NA	NA	0<br>*7*<br>*(13)*	NA	NA	NA	0	NA	NA	0<br>*41*<br>*(39)*	NA	NA	NA	NA	NA	NA	0	NA	NA	0<br>31<br>(74)<br>[a]
	Missing data for biochemical outcomes <10%<br>N (%)	0	0<br>*10*<br>*(10)*<br>[b]	0	0	0	0	0	0	0	0	0	1<br>*0*<br>*(0)*	1<br>*0*<br>*(0)*	0	1<br>1<br>(1)	0	0	0	0	0

Category	Criterium	Alcalar [29]	Carluccio [27]	Chin [30]	Dantas [31]	Dimopoulou [32]	Fathalla [33]	Gu [34]	Guo [35]	Hua [36]	Milian [38]	Nader [28]	Psaras [1]	Psaras [39]	Raappana [40]	Ritvonen [41]	Trepp [42]	Vandeva [43]	Vega-Beyhart [44]	Yamamoto [45]	Ye [46]
Outcomes	Missing data for PROMs <10%, N (%)	0	0<br>43 (30)	0	0	0	0	0	0<br>*115 (26)*	0	0	0	0	0	0	0	0	0	0	0<br>13 (18)	0
	Assay for measurement of GH, IGF-1, prolactin, cortisol, ACTH, FSH and LH reported and adequate[d]	0	0	0	0	0	0	1	0	0	0	0	0	0	0	0	0	0	0	0	0
	Biochemical results at follow-up described for the entire population, not just significant results	NA	NA	1	NA	NA	NA	0	NA	NA	0	NA	NA	NA	NA	NA	NA	1	NA	NA	0
General risk of bias score (%)[e]		43	43	31	43	43	29	50	50	13	6	7	29	29	43	36	29	38	29	36	6

General risk of bias assessment of included studies using a component approach based on Analyses of Observational Studies of Etiology (COSMO-E) and Risk of Bias In Non-randomised Studies (ROBINS) criteria*, FSH* Follicle stimulating hormone, *GH* growth hormone, *IGF-1* insulin-like growth factor-1, *ISOQOL* International Society for Quality of Life Research, *LH* lutheinizing hormone, *NA* not applicable, *PROMs* patient reported outcome measures.

[a] Second follow-up.

[b] Glucocorticoid exposure.

[c] Elevated prolactin level.

[d] Scoring: a point was given if the article described the method of hormone measurement adequately enough to assume there was no clinically relevant bias in diagnosis of each of the hormone producing pituitary adenomas. *Acromegaly*: either IGF-1 or GH determination was performed and described adequately and cutoffs were presented that comply with the guidelines at time of publication i.e. *IGF-1*: inter-assay and intra-assay coefficients were <8% and adequate reference values were presented for calculation of IGF-1. If IDS-iSYS was used, without presenting reference values, this was also considered adequate, as peer reviewed reference values have been published for this system. *GH*: adequate reference values were presented. *Cushing's Disease*: adequate reference values were used. *Prolactinoma*: system, gender and age specific cutoff values were presented for serum prolactin.

[e] General risk of bias score was calculated as total points divided by the number of applicable items, multiplied by 100%.

Supplement 9 – ISOQOL criteria per study

Section	Criterium	Alcalar [29]	Carluccio [27]	Chin [30]	Dantas [31]	Dimopoulou [32]	Fathalla [33]	Gu [34]	Guo [35]	Hua [36]	Milian [38]	Nader [28]	Psaras [1]	Psaras [39]	Raappana [40]	Ritvonen [41]	Trepp [42]	Vandeva [43]	Vega-Beyhart [44]	Yamamoto [45]	Ye [46]
Title and abstract	The PRO should be identified as an outcome in the abstract	1	1	1	1	1	1	1	1	1	1	1	1	0	1	1	1	1	1	1	1
	For 1° outcome: The title of the paper should be explicit as to the cohort study including a PRO	1	1	1	1	0	1	1	1	1	1	1	1	1	1	1	1	1	1	1	1
Introduction, background and objectives	The PRO hypothesis should be stated and should specify the relevant PRO domain(s) if applicable	0	0	0	0	1	0	0	0	0	0	0	1	0	0	0	0	0	0	0	0
	For 1° outcome: The introduction should contain a summary of PRO research that is relevant to the cohort study	0	0	1	0	0	1	0	1	1	1	0	1	0	0	1	1	1	1	0	0
	For 1° outcome: Additional details regarding the hypothesis should be provided, including the rationale for the selected domain(s), the expected direction(s) of change, and the time points for assessment	0	0	0	0	0	0	0	0	0	0	0	0	0	0	0	0	0	0	0	0
Outcomes registration	The mode of administration of the PRO tool and the methods of collecting data (e.g., telephone, other) should be described	1	1	0	0	0	0	0	1	1	0	1	0	0	1	1	1	0	0	0	0
	The rationale for choice of the PRO instrument used should be provided	0	1	1	0	0	1	1	1	1	1	1	1	1	0	1	1	1	0	1	0
	Evidence of PRO instrument validity and reliability should be provided or cited	0	1	1	1	1	1	0	1	1	0	0	1	0	0	1	1	1	1	1	1
	The intended HRQL data collection schedule should be provided	NA	NA	1	NA	NA	NA	1	NA	NA	1	NA	NA	NA	NA	NA	NA	1	NA	NA	NA
	PROs should be identified in the trial protocol; post hoc analyses should be identified	0	0	1	NA	1	0	0	NA	NA	1	NA	NA	1	0	0	NA	0	0	1	0
	The status of PRO as either a primary or secondary outcome should be stated	1	1	0	1	1	1	1	1	1	1	1	1	0	1	1	1	1	1	1	1
	For 1° outcome: A citation for the original development of the PRO instrument should be provided	0	1	1	0	1	1	1	1	1	1	1	1	1	1	1	1	1	0	0	1
	For 1° outcome: Windows for valid PRO responses should be specified and justified as being appropriate for the clinical context	0	0	0	0	0	0	0	1	0	0	0	0	0	0	0	0	0	0	0	0

Section	Criterium	Alcalar [29]	Carluccio [27]	Chin [30]	Dantas [31]	Dimopoulou [32]	Fathalla [33]	Gu [34]	Guo [35]	Hua [36]	Milian [38]	Nader [28]	Psaras [1]	Psaras [39]	Raappana [40]	Ritvonen [41]	Trepp [42]	Vandeva [43]	Vega-Beyhart [44]	Yamamoto [45]	Ye [46]
Sample size	For 1° outcome: There should be a power/sample size calculation relevant to the PRO based on a clinical rationale (e.g., anticipated effect size)	0	0	1	0	0	0	0	0	0	0	0	0	0	0	0	0	0	0	0	0
Statistical methods	There should be evidence of appropriate statistical analysis and tests of statistical significance for each PRO hypothesis tested	0	0	0	0	1	0	0	0	0	0	0	0	0	0	0	0	0	0	0	0
	Statistical approaches for missing data should be explicitly stated, and the extent of missing data should be stated	0	0	0	0	0	0	0	1	0	0	0	0	0	0	0	0	0	0	0	0
	For 1° outcome: The manner in which multiple comparisons have been addressed should be provided	0	0	1	0	1	0	0	0	0	0	0	0	0	0	0	0	0	0	0	0
Participant flow	A flow diagram or a description of the allocation of participants (if applicable) and those lost to follow-up should be provided for PROs specifically	1	1	1	NA	NA	NA	0	NA	NA	0	NA	NA	NA	NA	NA	NA	0	NA	NA	1
	The reasons for missing data should be explained	1	1	1	0	0	0	0	0	0	0	0	0	0	0	0	0	0	0	0	1
Baseline data	The study patients' characteristics should be described, including baseline PRO scores	1	0	1	1	1	1	1	1	1	1	1	1	1	1	1	1	1	1	1	0
Outcomes and estimation	The analysis of PRO data should account for survival differences between treatment groups if relevant	NA	NA	NA	NA	NA	NA	NA	NA	NA	NA	NA	NA	NA	NA	NA	NA	NA	NA	NA	NA
	Results should be reported for all PRO domains (if multi-dimensional) and items identified by the reference instrument (i.e., not just those that are statistically significant)	1	0	1	1	1	1	0	1	1	1	1	1	1	1	1	1	1	1	0	1
	The proportion of patients achieving predefined responder definitions should be provided where relevant	NA	NA	NA	NA	NA	NA	NA	NA	NA	NA	NA	NA	NA	NA	NA	NA	NA	NA	NA	NA
Limitations	The limitations of the PRO components of the study should be explicitly discussed	0	1	0	0	0	0	0	0	1	0	0	0	0	0	0	0	0	1	0	1
Generalizability	Generalizability issues uniquely related to the PRO results should be discussed, if applicable	0	0	0	0	0	0	0	0	0	0	0	0	0	0	0	0	0	1	0	1
Interpretation	The clinical significance of the PRO findings should be discussed	0	0	0	0	1	0	0	0	0	0	1	0	0	0	1	0	0	0	0	0
	The PRO results should be discussed in the context of the other clinical studies	1	1	1	1	1	1	1	1	1	1	1	1	0	1	1	1	1	1	1	1
Protocol	A copy of the instrument should be included if it has not been published previously (1 if published previously)	0	0	1	1	1	1	1	1	1	1	1	1	1	1	1	1	1	1	1	1
Percentage of items reported by study (%)		36	44	62	35	46	54	35	61	57	46	48	52	29	38	54	52	46	46	38	48

International Society for Quality of Life Research (ISOQOL) criteria modified for non-randomized controlled trials per study. *1° outcome* primary outcome, *NA* not applicable, *PRO* patient reported outcome, *PROM* patient reported outcome measure.

Supplement 10 -Tuebingen CD-25

First Author, year of publication	Nader [28][a]	
**Disease**	**CD**	
**Baseline**	**N (%), N=8**	
	**Mild**	**Severe**
Depression	4 (50%) =	1 (13%) =
Sexual activity	2 (25%) =	2 (25%) =
Environment	2 (25%) =	3 (38%) =
Eating behavior	3 (38%) =	1 (13%) =
Bodily restrictions	0 (0%) =	7 (88%) =
Cognition	1 (13%) =	3 (38%) =
Total	3 (38%) =	3 (38%) =

Tuebingen CD-25 scores for refractory patients with Cushing's Disease as reported by Nader et al. [28]. Mild: scores > percentile rank 84 of age- and gender-specific cut-off values. Severe: scores > percentile rank 95 of age- and gender-specific cut-off values. *CD* Cushing's Disease, *Tuebingen CD-25* Tuebingen Cushing's disease Quality of Life Inventory, = tested and no significant difference compared to CD patients in remission.
[a] Values estimated based on figure, absolute values were not presented.

Supplement 11 – EQ-5D-5L

First Author, year of publication	Guo [35]
**Disease**	**AC**
**Baseline**	**Mean/median/ percentage of patients checking "no problems" N=154**
VAS of EQ-5D	62.8 ± 21.6 =
mobility	0.018/0.000/81.8% =
Self-care	0.003/0.000/94.2% =
Usual activities	0.070/0.000/85.1% =
Pain/discomfort	0.106/0.058↑/12.3% ↓
Anxiety/depression	0.089/0.049↑/11.0%↓

EQ-5D-5L scores for refractory patients with acromegaly as reported by Guo et al**.** [35]. *AC* acromegaly, *EQ-5D-5L* 5-level EuroQoL-5, *VAS* visual analogue scale, ↓ significantly lower compared to acromegaly patients in remission ↑ significantly higher compared to acromegaly patients in remission, = tested and no significant difference compared to acromegaly patients in remission.

Supplement 12 - BDI

**First Author, year of publication**	**Alacalar [29]**	**Nader [28][b]**
**Disease**	**AC**	**CD**
**Baseline**	**Mean ± SD, N=8**	**N (%), N=8**
≤10 points		2 (25%) =
11-17 points		4 (50%) =
≥18 points		2 (25%) =
Total	18.9 ± 10.9 · [a]	

BDI scores for refractory patients with acromegaly or Cushing's disease per study. Included studies applied different cut-offs. Alcalar et al.: <17 points: absence of depression, ≥17 points: presence of depression. Nader et al.: ≤10 points: no depression, 11-17 points: mild to moderate depression, ≥18 points: severe depression. *AC* acromegaly, *CD* Cushing's Disease, *BDI* Beck Depression Inventory, *SD* standard deviation, · no P-value reported, = tested and no significant difference compared to patients in remission.

[a] No significant difference between refractory patients, those in remission and healthy controls (no post-hoc analysis was performed).

[b] Values estimated based on figure, absolute values were not presented.

Supplement 13 – SCL-90-R

First Author, year of publication	Psaras [39]	
**Disease**	**AC**	**CD**
**Baseline**	**Mean ± SD, N=14**	**Mean ± SD, N=5**
Somatization	58.5 ± 32.7 ·	57.2 ± 37.4 ·
Obsessive-Compulsive	58.5 ± 32.3 ·	70.2 ± 39.2 ·
Interpersonal Sensitivity	52.0 ± 33.3 ·	60.2 ± 34.6 ·
Depression	57.1 ± 33.9 ·	65.2 ± 42.8 ·
Anxiety	51.6 ± 31.8 ·	42.2 ± 36.6 ·
Hostility	59.5 ± 29.3 · [a]	60.4 ± 27.5 · [a]
Phobic Anxiety	61.5 ± 26.6 ·	53.8 ± 33.7 ·
Paranoid Ideation	55.2 ± 27.5 ·	55.0 ± 27.3 ·
Psychoticism	52.0 ± 29.9 ·	62.8 ± 28.5 · [a]
Global Severity Index	56.7 ± 34.3 ·	76.0 ± 19.6 ·
Positive symptom Total	56.9 ± 34.3 ·	62.0 ± 38.3 ·
Positive Symptom Distress Index	58.6 ± 28.1 ·	62.6 ± 34.0 ·

SCL-90-R scores for refractory patients with acromegaly and Cushing's Disease as reported by Psaras et al. [39]. *AC* acromegaly. *CD* Cushing's Disease, *SCL-90-R* Symptom Checklist-90-Revised, *SD* standard deviation,
· no P-value reported.
[a] Refractory patients scored significantly higher than healthy controls.

Supplement 14 - MBSRQ

**First Author, year of publication**	**Alcalar [29]**
**Disease**	**CD**
**Baseline**	**Mean ± SD, N=8**
Appearance evaluation	2.99 ± 0.49 ·
Appearance orientation	3.19 ± 0.54 ·
Fitness evaluation	2.79 ± 0.46 · [a]
Fitness orientation	2.77 ± 0.61 ·
Health evaluation	3.06 ± 1.08 · [a]
Health orientation	3.59 ± 0.83 ·
Body areas satisfaction	2.56 ± 0.86 · [a]
Mean item score	3.02 ± 0.33 · [a]

MBSRQ scores for refractory patients with Cushing's Disease as reported by Alcalar et al. [29]. *CD* Cushing's Disease, *MBSRQ* Multidimensional Body-Self relations Questionnaire, *SD* standard deviation,· no P-value reported.

[a] No post-hoc analysis was performed for refractory patients and those in remission, however there was a significant difference between refractory patients, those in remission and healthy controls.

References

1. Psaras, T., et al., *Are There Gender-Specific Differences Concerning Quality of Life in Treated Acromegalic Patients?* Experimental and Clinical Endocrinology & Diabetes, 2011. **119**(5): p. 300-305.
2. Webb, S.M., et al., *Acromegaly Quality of Life Questionnaire (ACROQOL) a new health-related quality of life questionnaire for patients with acromegaly: development and psychometric properties.* Clin Endocrinol (Oxf), 2002. **57**(2): p. 251-8.
3. Webb, S.M., et al., *Evaluation of health-related quality of life in patients with Cushing's syndrome with a new questionnaire.* European Journal of Endocrinology, 2008. **158**(5): p. 623-630.
4. Milian, M., et al., *Erratum: The development of the Tuebingen Cushing's disease quality of life inventory (Tuebingen CD-25). Part II: Normative data from 1784 healthy people (Clinical Endocrinology (2012) 76 (861-867)).* Clinical Endocrinology, 2013. **79(6)**: p. 901-903.
5. Milian, M., et al., *The development of the Tuebingen Cushing's disease quality of life inventory (Tuebingen CD-25). Part I: construction and psychometric properties.* Clin Endocrinol (Oxf), 2012. **76**(6): p. 851-60.
6. Flitsch, J., S. Spitzner, and D.K. Lüdecke, *Emotional disorders in patients with different types of pituitary adenomas and factors affecting the diagnostic process.* Exp Clin Endocrinol Diabetes, 2000. **108**(7): p. 480-5.
7. Herschbach, P., et al., *Development and psychometric properties of a disease-specific quality of life questionnaire for adult patients with growth hormone deficiency.* Eur J Endocrinol, 2001. **145**(3): p. 255-65.
8. *EuroQol--a new facility for the measurement of health-related quality of life.* Health Policy, 1990. **16**(3): p. 199-208.
9. Herdman, M., et al., *Development and preliminary testing of the new five-level version of EQ-5D (EQ-5D-5L).* Qual Life Res, 2011. **20**(10): p. 1727-36.
10. Hunt, S.M., et al., *A quantitative approach to perceived health status: a validation study.* J Epidemiol Community Health, 1980. **34**(4): p. 281-6.
11. Ware, J.E., Jr. and C.D. Sherbourne, *The MOS 36-item short-form health survey (SF-36). I. Conceptual framework and item selection.* Med Care, 1992. **30**(6): p. 473-83.
12. Hays, R.D., C.D. Sherbourne, and R.M. Mazel, *The RAND 36-Item Health Survey 1.0.* Health Econ, 1993. **2**(3): p. 217-27.
13. Ware, J., Jr., M. Kosinski, and S.D. Keller, *A 12-Item Short-Form Health Survey: construction of scales and preliminary tests of reliability and validity.* Med Care, 1996. **34**(3): p. 220-33.
14. Sintonen, H., *The 15D instrument of health-related quality of life: properties and applications.* Ann Med, 2001. **33**(5): p. 328-36.
15. Clonninger, C.R., *The Tridimensional Personality Questionnaire, Version iu. St. Louis.* 1987.
16. Weyers, P., H. Krebs, and W. Janke, *Reliability and construct validity of the German version of Cloninger's Tridimensional Personality Questionnaire.* Personality and Individual Differences, 1995. **19**(6): p. 854-861.
17. Ruch, W., *Die revidierte Fassung des Eysenk Personality Questionnaire und die Konstruktion des Deutchen EPQR bzw. EPQ-RK.* Zeitschrift für Differentielle und Diagnostische Psychologie, 1999. **20**: p. 1-14.
18. Beck, A.T., et al., *An inventory for measuring depression.* Arch Gen Psychiatry, 1961. **4**: p. 561-71.
19. Eaton, W.W., et al., *Center for Epidemiologic Studies Depression Scale: Review and revision (CESD and CESD-R).* The Use of Psychological Testing for Treatment Planning and Outcomes Assessment, 2004: p. 363-377.
20. Radloff, L.S., *The CES-D scale: a self-report depression scale for research in the general population. Applied Psychological Measurement.* 1977: p. 1 3, 85-401.
21. Zigmond, A.S. and R.P. Snaith, *The hospital anxiety and depression scale.* Acta Psychiatr Scand, 1983. **67**(6): p. 361-70.
22. Derogatis, L.R., *SCL-90-R: Administration, Scoring and Procedures: Manual II.* . Psychology & Health, 1983. **7**(06).
23. Cooper, P.J., et al., *The development and validation of the body shape questionnaire.* International Journal of Eating Disorders, 1987. **6**(4): p. 485-494.
24. Stunkard, A.J., T. Sørensen, and F. Schulsinger, *Use of the Danish Adoption Register for the study of obesity and thinness.* Res Publ Assoc Res Nerv Ment Dis, 1983. **60**: p. 115-20.
25. Raich, R.M., J.T. Clarasó, and M. Figueras, *Estudio de la imagen corporal y su relación con el deporte en una muestra de estudiantes universitarios.* Análisis y modificación de conducta, 1996. **22**(85): p. 603-626.
26. Cash, T.F., *User’s manual for the multidimensional body-self relations questionnaire.* Norfolk, VA: Old Dominion University, 2000.
27. Carluccio, A., et al., *Predictors of quality of life in 102 patients with treated Cushing's disease.* Clin Endocrinol (Oxf), 2015. **82**(3): p. 404-11.
28. Nader, S., et al., *Health-related Quality of Life in Patients After Treatment of Cushing's Disease.* Experimental and Clinical Endocrinology & Diabetes, 2016. **124**(3): p. 187-191.
29. Alcalar, N., et al., *Evaluation of depression, quality of life and body image in patients with Cushing's disease.* Pituitary, 2013. **16**(3): p. 333-40.
30. Chin, S.O., et al., *Change in quality of life in patients with acromegaly after treatment with octreotide LAR: first application of AcroQoL in Korea.* Bmj Open, 2015. **5**(6).
31. Elias Dantas, R.A., et al., *Physical activities in daily life and functional capacity compared to disease activity control in acromegalic patients: Impact in self-reported quality of life.* Arquivos Brasileiros de Endocrinologia e Metabologia, 2013. **57(7)**: p. 550-557.
32. Dimopoulou, C., et al., *Increased Prevalence of Anxiety-Associated Personality Traits in Patients with Cushing's Disease: A Cross-Sectional Study.* Neuroendocrinology, 2013. **97**(2): p. 139-145.
33. Fathalla, H., et al., *Endoscopic transphenoidal surgery for acromegaly improves quality of life.* Can J Neurol Sci, 2014. **41**(6): p. 735-41.

34. Gu, J., et al., *Quality of Life in Patients with Acromegaly before and after Transsphenoidal Surgical Resection.* Int J Endocrinol, 2020. **2020**: p. 5363849.
35. Guo, X.P., et al., *Quality of Life and its Determinants in Patients With Treated Acromegaly: A Cross-Sectional Nationwide Study in China.* Journal of Clinical Endocrinology & Metabolism, 2021. **106**(1): p. 211-225.
36. Hua, S.C., Y.H. Yan, and T.C. Chang, *Associations of remission status and lanreotide treatment with quality of life in patients with treated acromegaly.* European Journal of Endocrinology, 2006. **155**(6): p. 831-837.
37. Leães, C.G.S., et al., *Assessment of Anthropometric and Physical Health Indicators before and after Pituitary Surgery in Patients with Nonfunctioning Pituitary Adenomas, Acromegaly, and Cushing Disease.* Indian J Endocrinol Metab, 2019. **23**(4): p. 473-479.
38. Milian, M., et al., *Health-related quality of life and psychiatric symptoms improve effectively within a short time in patients surgically treated for pituitary tumors--a longitudinal study of 106 patients.* Acta Neurochir (Wien), 2013. **155**(9): p. 1637-45; discussion 1645.
39. Psaras, T., et al., *Predictive factors for neurocognitive function and Quality of Life after surgical treatment for Cushing's disease and acromegaly.* Journal of Endocrinological Investigation, 2011. **34**(7): p. E168-E177.
40. Raappana, A., et al., *Long-term health-related quality of life of surgically treated pituitary adenoma patients: a descriptive study.* ISRN Endocrinol, 2012. **2012**: p. 675310.
41. Ritvonen, E., et al., *Normal long-term health-related quality of life can be achieved in patients with functional pituitary adenomas having surgery as primary treatment.* Clinical Endocrinology, 2015. **82(3)**: p. 412-421.
42. Trepp, R., et al., *Assessment of quality of life in patients with uncontrolled vs. controlled acromegaly using the Acromegaly Quality of Life Questionnaire (AcroQoL).* Clinical Endocrinology, 2005. **63**(1): p. 103-110.
43. Vandeva, S., et al., *Disease control and treatment modalities have impact on quality of life in acromegaly evaluated by Acromegaly Quality of Life (AcroQoL) Questionnaire.* Endocrine, 2015. **49**(3): p. 774-782.
44. Vega-Beyhart, A., et al., *Quality of life is significantly impaired in both secretory and non-functioning pituitary adenomas.* Clinical Endocrinology, 2019. **90**(3): p. 457-467.
45. Yamamoto, N., et al., *The Effect of Aging on Quality of Life in Acromegaly Patients Under Treatment.* Frontiers in Endocrinology, 2022. **13**.
46. Ye, V.C. and R. Akagami, *Perioperative Quality of Life in Cushing's Disease.* Can J Neurol Sci, 2017. **44**(1): p. 69-77.
